# Brain morphological variability between whites and African Americans: the importance of racial identity in brain imaging research

**DOI:** 10.3389/fnint.2023.1027382

**Published:** 2023-12-21

**Authors:** Daniel Atilano-Barbosa, Fernando A. Barrios

**Affiliations:** Institute of Neurobiology, National Autonomous University of Mexico, Juriquilla, Mexico

**Keywords:** brain volumetry, cortical thickness, cortical surface area, racial identity, MRI, Human Connectome Project

## Abstract

In a segregated society, marked by a historical background of inequalities, there is a consistent under-representation of ethnic and racial minorities in biomedical research, causing disparities in understanding genetic and acquired diseases as well as in the effectiveness of clinical treatments affecting different groups. The repeated inclusion of small and non-representative samples of the population in neuroimaging research has led to generalization bias in the morphological characterization of the human brain. A few brain morphometric studies between Whites and African Americans have reported differences in orbitofrontal volumetry and insula cortical thickness. Nevertheless, these studies are mostly conducted in small samples and populations with cognitive impairment. For this reason, this study aimed to identify brain morphological variability due to racial identity in representative samples. We hypothesized that, in neurotypical young adults, there are differences in brain morphometry between participants with distinct racial identities. We analyzed the Human Connectome Project (HCP) database to test this hypothesis. Brain volumetry, cortical thickness, and cortical surface area measures of participants identified as *Whites* (*n* = 338) or *African Americans* (*n* = 56) were analyzed. Non-parametrical permutation analysis of covariance between these racial identity groups adjusting for age, sex, education, and economic income was implemented. Results indicated volumetric differences in choroid plexus, supratentorial, white matter, and subcortical brain structures. Moreover, differences in cortical thickness and surface area in frontal, parietal, temporal, and occipital brain regions were identified between groups. In this regard, the inclusion of sub-representative minorities in neuroimaging research, such as African American persons, is fundamental for the comprehension of human brain morphometric diversity and to design personalized clinical brain treatments for this population.

## 1 Introduction

Human population studies are contributing to understand variability in the prevalence of diseases, treatment response, risk factors, and relationships between genetic and environmental outcomes between diverse societal groups (Falk et al., 2013; Batai et al., 2021). Accordingly, human brain morphological variability has been robustly associated with individual genetic ancestry (Fan et al., 2015) and sociocultural influences (Holz et al., 2014; Noble et al., 2015). One of the methodologies used for the characterization of the human brain has been morphological neuroimaging analysis, which consists of the implementation of computational analysis methods of brain magnetic resonance imaging (MRI), aimed to identify the structural characteristics of the brain, highlighting analysis of volume and area, such as cortical surface area and cortical thickness (Mietchen and Gaser, 2009). Brain volumetry is a measure that includes surface area and cortical thickness (Panizzon et al., 2009); the former being a parameter of cortical folding and gyrification (Rakic, 2009) and the latter a parameter of density and dendritic arborization (Huttenlocher, 1990).

Neuroimaging studies have been implemented to identify brain morphometric differences due to educational level (Ho et al., 2011), socioeconomic status (Farah, 2017), gender, and age (Smith et al., 2007; Takahashi et al., 2011). Nevertheless, few neuroimaging studies are designed to explore brain morphometric differences related to racial identity. In this sense, it has been reported that African American persons diagnosed with hypertension and cognitive impairment, commonly referred to as a decline in memory and cognition performance, have lower insular thickness compared to White persons with the same diagnosis (Chand et al., 2017). Moreover, Isamah et al. (2010) implemented a volumetric analysis using magnetic resonance imaging (MRI) in neurotypical White and African American persons. After controlling for variables such as age, sex, years of education, and total brain volume, they reported that African American participants had a greater brain volume of the left orbitofrontal cortex than White participants. These authors agree that morphometric studies in populations with diverse racial identifications will reduce the under-representation of ethnic minorities as well as the comprehension of the influence of these variables on the differentiation in specific brain structures and the prevalence of neuropsychiatric diseases among different populations.

Racial identity has generally been used as a demographic variable and not as a variable of interest in neuroimaging research, which contributes to generalization bias of brain findings based on persons with high educational and socioeconomic status belonging to majority racial groups (Falk et al., 2013; Rouan et al., 2021). Furthermore, studies including minority racial groups are mainly implemented in small samples and in populations with cognitive impairment (Isamah et al., 2010; Chand et al., 2017). Thus, our study aimed to identify morphological brain variability among distinct racial identities in a representative sample of neurotypical young adults. We analyzed brain morphometric data from the Human Connectome Project (HCP) (van Essen et al., 2012). Our selection criteria indicate that *White* and *African American* racial identities were the most representative samples in the HCP database. In this regard, we expect to identify differences in brain morphometry between people identified as *Whites* or *African Americans*.

## 2 Methods

In order to access participants’ racial identity information, all authors accepted the terms of data used to access restricted data of the HCP database. After the request was accepted by the WU-Minn HCP Consortium, the database from 1,206 participants was downloaded from the ConnectomeDB, a web-based user interface from the HCP (Hodge et al., 2015). Apart from racial identity information, restricted data included demographic, clinical, psychiatric, and morphometric brain information for each participant. Data were filtered to exclude participants with psychiatric symptoms, substance use and abuse disorders, endocrine disorders, irregular menstrual cycles, neurological abnormalities, and technical issues in the acquisition or preprocessing of their structural brain images. In the filtered database, participants identified as Hispanics were discarded due to unbalanced sample representation between the selected racial identity groups (Hispanic-Whites = 22, Hispanic-African Americans *n* = 1). Beyond this classification, ethnic identity was not considered for further analysis. Racial identity categories referred to Whites and African Americans were taken from the HCP demographic data based on the NIH Toolbox and U.S. Census classification.^1^

Three hundred thirty-eight participants identified as *Whites* [*M*_age(y)_ = 29.12, SD = ±3.60_,_ *M*_education(y)_ = 15.15, SD = ±1.69] and 56 subjects identified as *African Americans* [*M*_age(y*)*_ = 29.25, SD = ±3.62, *M*_education(y)_ = 14.41, SD = ±1.90] satisfied the inclusion criteria from the filtering process of the original HCP database. Although age [*t*_(392)_ = −0.2533, *p =* 0.800] was not significantly different, years of education between groups resulted in significant differences [*t*_(70.96)_ = 2.760, *p =* 0.007]. Moreover, three participants identified as *Whites* were excluded from the permutation analysis because of missing education and economic income information (see Table 1).

**Table 1:** Descriptive results between African Americans and Whites.

	***N***	**Racial identity**		**Value of *p***^**2**^
		**African Americans**, *N* = 56^1^	**Whites**, *N* = 338^1^	
Age (years)	394	29.25 (3.62)	29.12 (3.60)	0.801
Sex	394			
Male		23 (41%)	190 (56%)	
Female		33 (59%)	148 (44%)	
Education (years)	392	14.41 (1.90)	15.15 (1.69)	0.007
Economic Income (US$)	391			
<$10,000		7 (12%)	12 (3.6%)	
10K-19999		10 (18%)	22 (6.6%)	
20K-29999		9 (16%)	34 (10%)	
30K-39999		10 (18%)	32 (9.6%)	
40K-49999		5 (8.9%)	39 (12%)	
50K-74999		8 (14%)	76 (23%)	
75K-99999		1 (1.8%)	53 (16%)	
> = 100,000		6 (11%)	67 (20%)	
*Missing*		0	3	

^1^Mean (SD) of age and education in years. Frequencies (*n*) and percentages (%) of economic income ranges in US dollars. ^2^Welch two sample t-test of age and education between African Americans and Whites (*p* < 0.05).

Summary statistics of FreeSurfer morphometric measures (volume, cortical surface area, and cortical thickness) from the HCP database previously processed by HCP investigators were analyzed (Glasser et al., 2013). These preprocessing methods consist of a *PreFreeSurfer* pipeline, which was implemented to preprocess high-resolution T1w and T2w (weighted) brain images (0.7 mm thickness) for each participant to produce an undistorted “native” structural volume space. The pipeline aligned the T1w and T2w brain images, executed a B1 (bias field) correction for each volume, and co-registered the participant’s undistorted structural volume space to MNI space. Subsequently, a *Freesurfer* pipeline was executed to divide the native volume into cortical and subcortical parcels, reconstruct white and pial cortical surfaces, and perform the standardized FreeSurfer’s folding-based surface mapping to their surface atlas (fsaverage) (Glasser et al., 2013). Volumetric, cortical thickness, and surface area brain measures were grouped by participants’ racial identity—*Whites* or *African Americans*. Before applying statistical analysis, volumetric results for each participant were standardized by dividing the raw volumetric scores by intracranial volume (ICV). Due to unbalanced samples between groups, ANCOVA permutation analyses adjusting for *age, sex, education,* and *economic income* were implemented to identify differences between groups for each brain morphometric measure. The estimation of value of *p*s was based on the criterion in which iteration stopped when the estimated standard error of the estimated proportion of the value of *p* was less than one-thousandth of the estimated value of *p* (Anscombe, 1953). A maximum of 5,000 iterations were selected for the analysis. Adjustment of value of *p*s for multiple comparisons were implemented by the family-wise error (FWE) rate method (Holm, 1979). Due to sample imbalance, a subsample selection from the filtered database was implemented, based on the criteria that participants from the higher sample size group (*Whites*) were similarly paired in *age, sex, education,* and *economic income* to the low sample size group (African Americans) (see Supplementary Table S4). ANCOVA permutation analyses corrected for multiple comparisons (FWE) on the same morphological parameters described above were implemented for this subsample.

The ordering of the database, data filtering, and statistical analysis was carried out in the programming language R version 3.6.3 mounted on the RStudio software version 1.2.5033. ANCOVA permutation analysis was implemented by the aovp function of the *lmperm* package in R (Wheeler and Torchiano, 2016). The pipeline used for the statistical analysis can be consulted at https://github.com/Daniel-atilano/HCP_structural_analysis.git.

## 3 Results

### 3.1 Brain volumetry differences between groups

**Figure 1:**
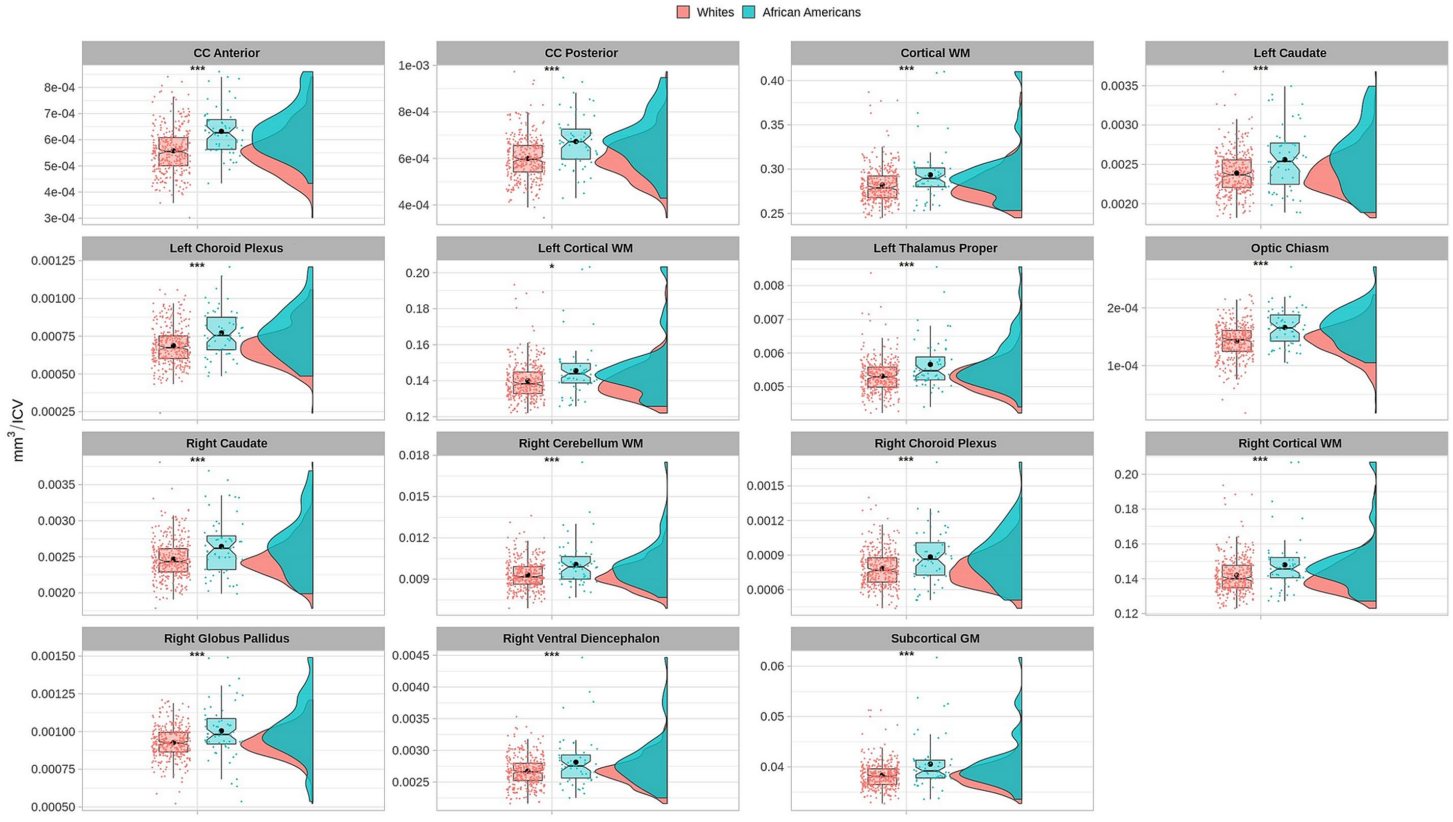
Permutational ANCOVA brain volumetric results between Whites and African Americans with significant differences after applying multiple comparison correction test (FWER). Brain volumetry is standardized as the ratio of cubic millimeters/intracranial volume (mm^3^/ICV). CC anterior: anterior subregion of corpus callosum. CC posterior: posterior subregion of corpus callosum. WM, white matter. GM, gray matter. Asterisks (***) indicate significant results at a value of *p* of <0.001 and (*) a value of *p* of <0.05.

Volume comparisons resulted in significant differences in cortical and subcortical brain structures (see Table 2 and Figure 1).

**Table 2:** ANCOVA permutation volumetric brain results between African Americans and Whites adjusting for *age, sex, education,* and *economic income.*

Volumetric measure (mm^3^/ICV)	African Americans, *N* = 56 Mean (SD)	Whites, *N* = 335 Mean (SD)	df	MSS	Iteration	Value of *p*	*p*-adjust value
Subcortical gray matter	0.04055 (0.005034)	0.03834 (0.002698)	1	0.00011	5,000	2e-16	0.0000***
Cortical white matter, L	0.1456 (0.01530)	0.1400 (0.01035)	1	0.00112	5,000	0.0004	0.0148*
Cortical white matter, R	0.148 (0.01591)	0.142 (0.01041)	1	0.00133	5,000	2e-16	0.0000***
Total cortical white matter	0.2936 (0.03118)	0.2820 (0.02072)	1	0.00491	5,000	2e-16	0.0000***
Thalamus proper, L	0.005662 (0.0007313)	0.005314 (0.0004950)	1	2.97e-06	5,000	2e-16	0.0000***
Caudate, L	0.002561 (0.0003973)	0.002389 (0.0002699)	1	6.66e-07	5,000	2e-16	0.0000***
Choroid plexus, L	0.0007718 (0.0001564)	0.0006879 (0.0001265)	1	2.74e-07	5,000	2e-16	0.0000***
Cerebellum white matter, R	0.010071 (0.001618)	0.009315 (0.001034)	1	2.31e-05	5,000	2e-16	0.0000***
Caudate, R	0.002643 (0.0003947)	0.002467 (0.0002746)	1	7.50e-07	5,000	2e-16	0.0000***
Pallidum, R	0.001005 (0.0001865)	0.000927 (0.0001037)	1	1.76e-07	5,000	2e-16	0.0000***
Ventral diencephalon, R	0.002813 (0.0003966)	0.002673 (0.0002312)	1	6.68e-07	5,000	2e-16	0.0000***
Choroid plexus, R	0.0008843 (0.0002290)	0.0007852 (0.0001559)	1	4.19e-07	5,000	2e-16	0.0000***
Optic chiasm	0.0001660 (3.156e-05)	0.0001423 (2.936e-05)	1	2.83e-08	5,000	2e-16	0.0000***
Posterior corpus callosum	0.0006727 (1.129e-04)	0.0005998 (9.114e-05)	1	1.44e-07	5,000	2e-16	0.0000***
Anterior corpus callosum	0.0006323 (9.232e-05)	0.0005577 (8.614e-05)	1	1.75e-07	5,000	2e-16	0.0000***

(***) Significant results at a value of *p* of < **0**.001 and (*) a value of *p* of < 0.05 when multiple comparison correction test (FWER) was applied. R, Right Hemisphere. L, Left Hemisphere. MSS, Mean sum of squares. Brain volumetry is standardized as the ratio of cubic millimeters / intracranial volume (mm^3^/ICV).

**Figure 2:**
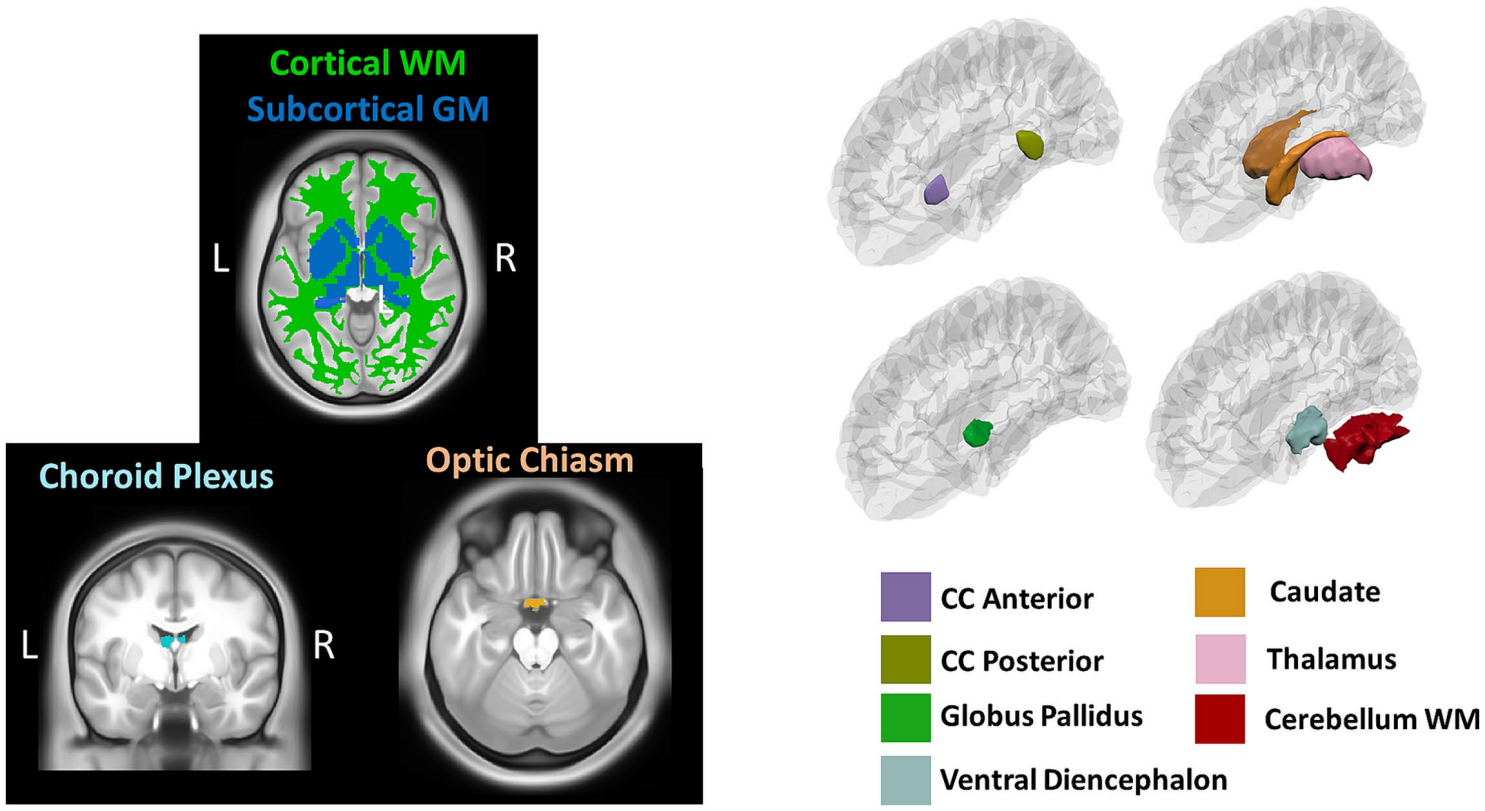
Brain regions representing volumetric differences between Whites and African Americans. CC, corpus callosum; WM, white matter. GM, gray matter. Brain images were created with *BrainPainter* software (Marinescu et al., 2019).

Volumetric measures were obtained from a volume-based stream where MRI volumes are labeled to classify subcortical and cortical tissues based on subject-independent probabilistic atlas and subject-specific measured values of voxels. Anatomical visualization of brain regions with significant statistical volumetric differences is represented in Figure 2.

Similar results were found on the paired subsample volumetric measures; nevertheless, after multiple comparisons correction (FWER), the bilateral and total cortical white matter, the left cerebellar white matter, the bilateral thalamus, and the anterior section of the corpus callosum maintain significant differences (see Supplementary Table S5).

### 3.2 Differences in cortical thickness between groups

**Figure 3:**
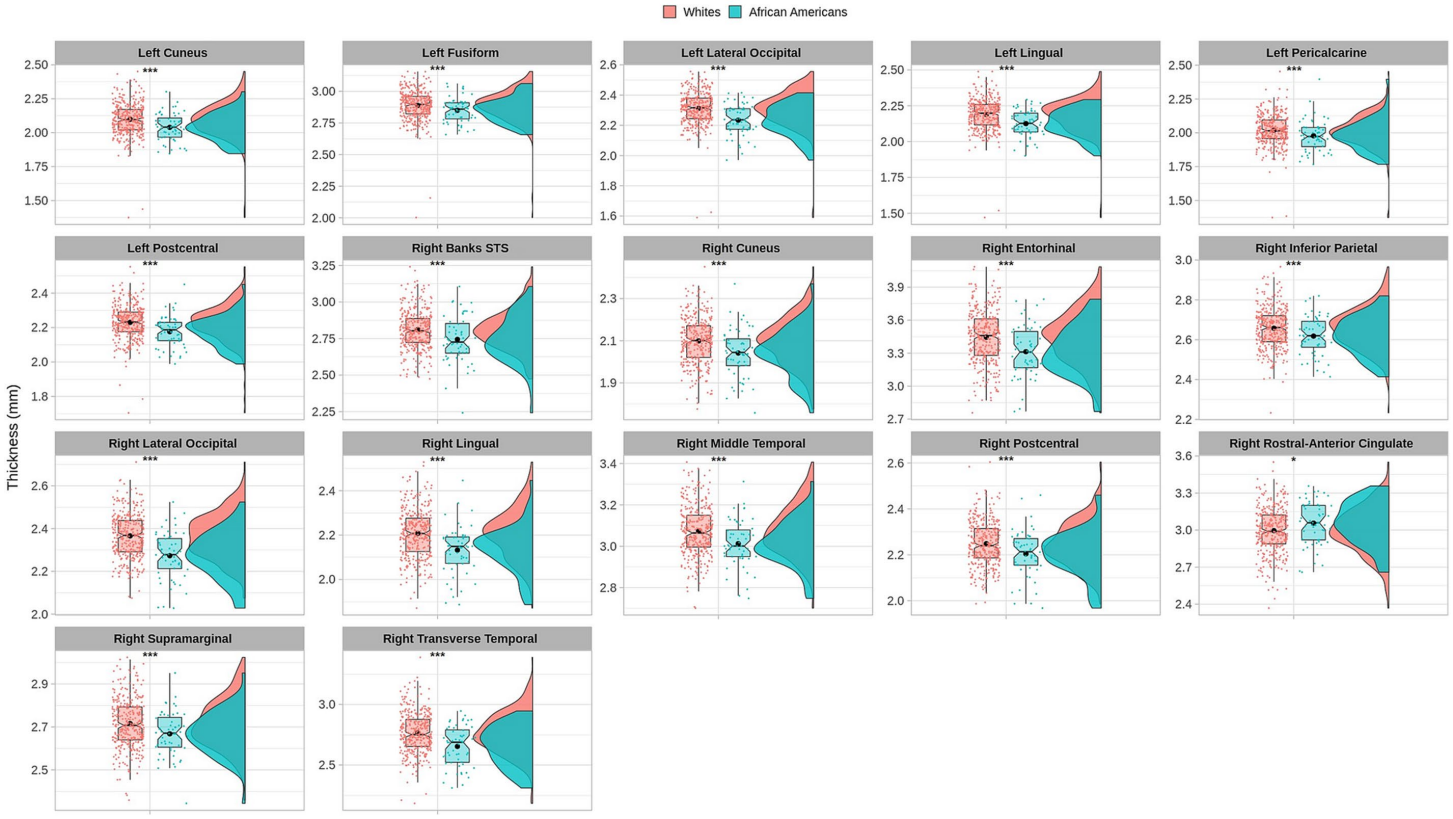
Permutational ANCOVA cortical thickness (mm) results between Whites and African Americans with significant differences after applying multiple comparison correction tests (FWER). Asterisks (***) indicate significant results at a value of *p* of <0.001 and (*) a value of *p* of <0.05.

Cortical thickness results indicated significant differences in frontal, temporal, parietal, and occipital brain regions (see Table 3 and Figure 3).

**Table 3:** Permutational ANCOVA cortical thickness results between African Americans and Whites adjusting for *age, sex, education,* and *economic income.*

Brain region (mm)	African Americans, *N* = 56 Mean (SD)	Whites, *N* = 335 Mean (SD)	df	MSS	Iteration	Value of *p*	*p*-adjust value
Banks of superior temporal sulcus, R	2.744 (0.1603)	2.810 (0.1342)	1	0.21868	5,000	2e-16	0.0000***
Cuneus cortex, L	2.040 (0.1011)	2.099 (0.1214)	1	0.20477	5,000	2e-16	0.0000***
Cuneus cortex, R	2.042 (0.1147)	2.099 (0.1105)	1	0.17220	5,000	2e-16	0.0000***
Entorhinal cortex, R	3.313 (0.2290)	3.445 (0.2412)	1	0.64413	5,000	2e-16	0.0000***
Fusiform gyrus, L	2.850 (0.09736)	2.889 (0.12094)	1	0.06748	5,000	2e-16	0.0000***
Inferior parietal cortex, R	2.618 (0.09668)	2.657 (0.10264)	1	0.09692	5,000	2e-16	0.0000***
Lateral occipital cortex, L	2.234 (0.1040)	2.312 (0.1099	1	0.27342	5,000	2e-16	0.0000***
Lateral occipital cortex, R	2.273 (0.1102)	2.366 (0.1036)	1	0.35269	5,000	2e-16	0.0000***
Lingual gyrus, L	2.125 (0.09406)	2.189 (0.11238)	1	0.14523	5,000	2e-16	0.0000***
Lingual gyrus, R	2.132 (0.1075)	2.208 (0.1101)	1	0.23351	5,000	2e-16	0.0000***
Middle temporal gyrus, R	3.013 (0.1111)	3.074 (0.1211)	1	0.13934	5,000	2e-16	0.0000***
Pericalcarine cortex, L	1.978 (0.1141)	2.020 (0.1170)	1	0.11471	5,000	2e-16	0.0000***
Postcentral gyrus, L	2.176 (0.09014)	2.230 (0.10179)	1	0.12848	5,000	2e-16	0.0000***
Postcentral gyrus, R	2.204 (0.10093)	2.248 (0.09787)	1	0.08111	5,000	2e-16	0.0000***
Rostral anterior Cingulate cortex, R	3.056 (0.1737)	2.996 (0.1853)	1	0.18686	5,000	0.0006	0.0312*
Supramarginal gyrus, R	2.668 (0.1017)	2.715 (0.1152)	1	0.14505	5,000	2e-16	0.0000***
Transverse temporal cortex, R	2.653 (0.1645)	2.765 (0.1709)	1	0.50098	5,000	2e-16	0.0000***

(***) Significant results at a value of *p* of < **0**.001 and (*) a value of *p* of < 0.05 when multiple comparison correction test (FWER) was applied. R, right hemisphere. L, left hemisphere. MSS, mean sum of squares.

**Figure 4:**
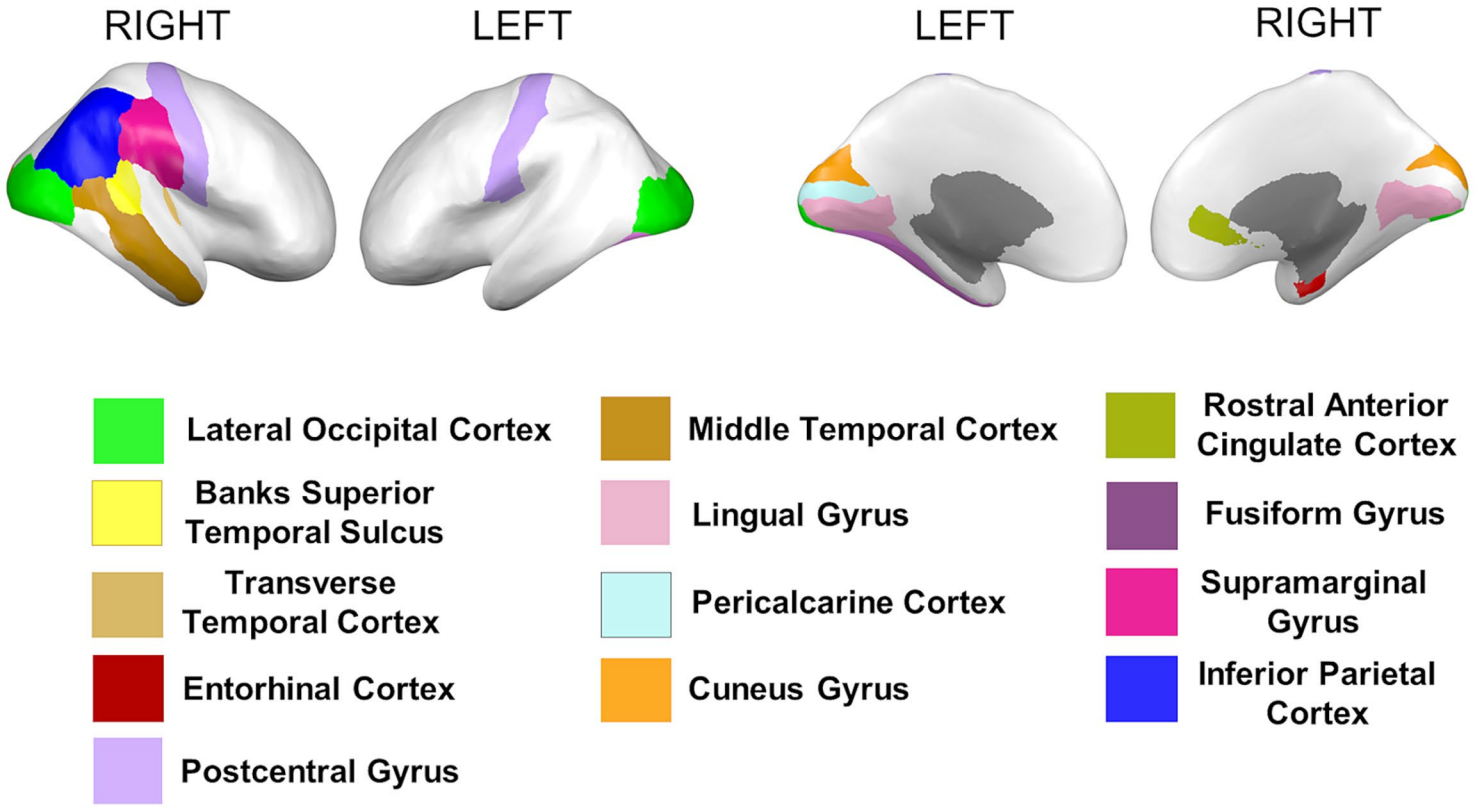
Brain regions representing cortical thickness differences between Whites and African Americans. Brain images were created with *BrainPainter* software (Marinescu et al., 2019).

Cortical thickness measures were obtained from the mean distance between the white and the pial surfaces of the cortex. Anatomical visualization of brain regions with significant statistical cortical thickness differences is represented in Figure 4.

Similar results were found on the paired subsample cortical thickness measures; nevertheless, after multiple comparisons correction (FWER), the right banks of the superior temporal sulcus, left cuneus cortex, the right middle temporal gyrus, the right supramarginal gyrus, and the right lateral occipital cortex maintain significant differences (see Supplementary Table S6).

### 3.3 Differences in cortical surface areas between groups

**Figure 5:**
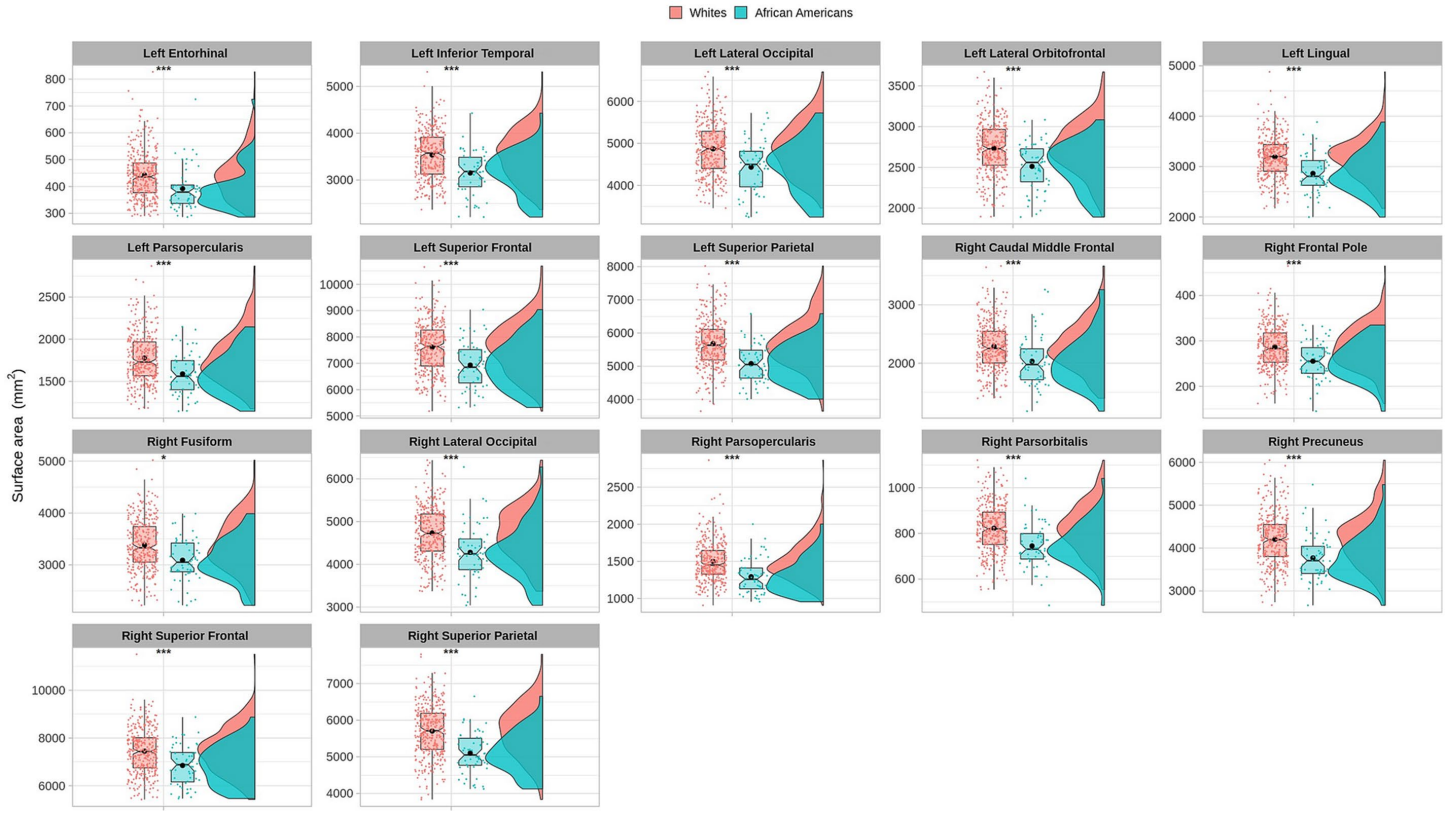
Permutational ANCOVA cortical surface area (mm^2^) results between Whites and African Americans with significant differences after applying the multiple comparison correction test (FWER). Asterisks (***) indicate significant results at a value of *p* of <0.001 and (*) a value of *p* of <0.05.

Cortical surface results indicated significant differences in frontal, temporal, parietal, and occipital brain regions (see Table 4 and Figure 5).

**Table 4:** Permutational ANCOVA surface cortical area results between African Americans and Whites adjusting for *age, sex, education,* and *economic income.*

Brain region (mm^2^)	African Americans, *N* = 56 Mean (SD)	Whites, *N* = 335 Mean (SD)	df	MSS	Iteration	Value of *p*	*p*-adjust value
Caudal middle frontal gyrus, R	2031.68 (430.703)	2289.21 (412.912)	1	1,313,656	5,000	2e-16	0.0000***
Entorhinal cortex, L	392.036 (79.9331)	442.269 (89.4814)	1	58,967	5,000	2e-16	0.0000***
Frontal pole, R	255.232 (41.2782)	286.684 (46.4144)	1	22,534	5,000	2e-16	0.0000***
Fusiform gyrus, R	3091.07 (409.400)	3385.21 (485.103)	1	979,123	5,000	0.0004	0.0208*
Inferior temporal gyrus, L	3151.93 (459.970)	3535.21 (514.899)	1	2,869,915	5,000	2e-16	0.0000***
Lateral occipital cortex, L	4432.77 (606.078)	4869.55 (621.979)	1	4,366,717	5,000	2e-16	0.0000***
Lateral occipital cortex, R	4281.59 (629.353)	4734.67 (607.326)	1	3,922,367	5,000	2e-16	0.0000***
Lateral orbitofrontal cortex, L	2511.55 (288.722)	2733.41 (318.287)	1	703,296	5,000	2e-16	0.0000***
Lingual gyrus, L	2863.04 (396.938)	3190.75 (415.092)	1	2,569,762	5,000	2e-16	0.0000***
Parsopercularis, L	1589.21 (246.493)	1777.46 (293.377)	1	774,206	5,000	2e-16	0.0000***
Parsopercularis, R	1292.12 (226.984)	1498.99 (265.773)	1	1,301,307	5,000	2e-16	0.0000***
Parsorbitalis, R	745.054 (95.406)	822.463 (106.355)	1	90,705	5,000	2e-16	0.0000***
Precuneus cortex, R	3770.21 (527.036)	4201.71 (609.588)	1	2,710,670	5,000	2e-16	0.0000***
Superior frontal gyrus, L	6931.23 (868.670)	7610.22 (962.951)	1	7,349,511	5,000	2e-16	0.0000***
Superior frontal gyrus, R	6849.00 (828.756)	7445.82 (928.692)	1	4,132,493	5,000	2e-16	0.0000***
Superior parietal cortex, L	5080.77 (565.338)	5680.33 (731.003)	1	6,302,632	5,000	2e-16	0.0000***
Superior parietal cortex, R	5100.62 (558.739)	5704.45 (699.319)	1	6,535,061	5,000	2e-16	0.0000***

(***) Significant results at a value of *p* of < 0.001 and (*) a value of *p* of < 0.05 when multiple comparison correction test (FWER) was applied. R, right hemisphere. L, left hemisphere. MSS, mean sum of squares.

**Figure 6:**
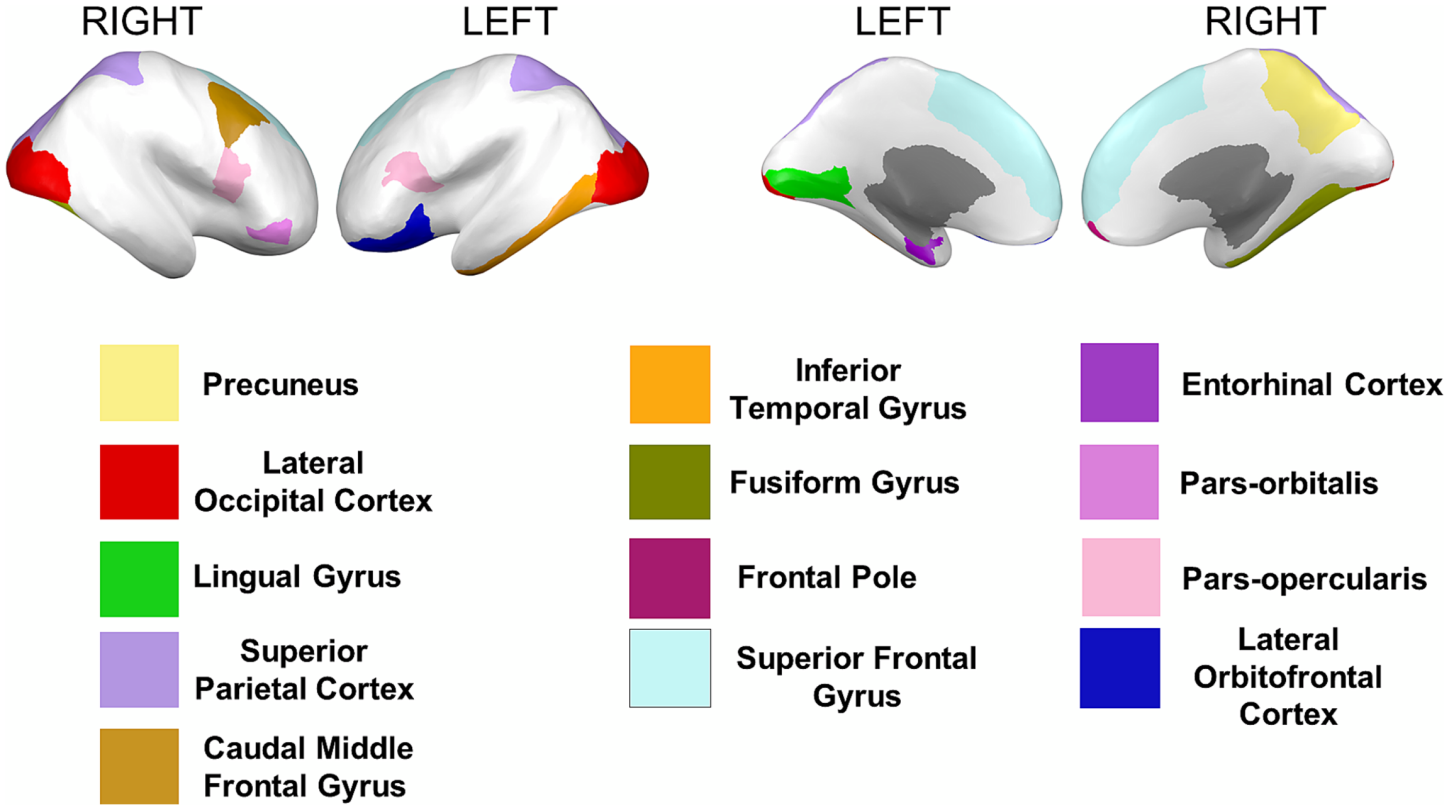
Brain regions representing cortical surface area differences between Whites and African Americans. Brain images were created with *BrainPainter* software (Marinescu et al., 2019).

Cortical surface measures were obtained from the sum of areas of triangles from the tessellation of the brain surface. Anatomical visualization of brain regions with significant statistical cortical surface area differences is represented in Figure 6.

Similar results were found on the paired subsample cortical surface area measures; nevertheless, none of the brain regions presents significant differences after applying multiple comparisons correction (FWER) (see Supplementary Table S7).

## 4 Discussion

Social, educational, and economic inequalities have impacted the health and human rights of ethnic and racial minorities, causing their under-representation in biomedical studies, leading to bias in the effectiveness of clinical treatments and misconceptions of genetic and environmental diseases affecting these groups (Konkel, 2015). According to some estimates, reducing such disparities would have saved the United States more than $ 1.2 billion in direct and indirect medical costs (Laveist et al., 2011). Even though the White non-Hispanic population has been steadily declining in recent years, African Americans and Hispanic/Latinos only represent 5 and 1% of participation in human research, while Whites represent over 70% (Ricard et al., 2022). In this regard, racial/ethnic identity is essential to contextualize neurophysiological and neuroimaging results on structural inequities in society (Harnett et al., 2023). In neuroimaging research, this under-representation bias may be responsible for the reproducibility, generalizability, external validity, and inference crisis in brain research, which exacerbates the disparities and inequalities of minorities in neuroscience (Falk et al., 2013; Dotson et al., 2020). Data sharing and open access to multimodal brain imaging in consortium repositories have been proposed as research opportunities to diminish racial disparities and methodological bias (Falk et al., 2013; Weinberger et al., 2020). Consequently, some advantages of using the HCP database are its public accessibility, a large ethnic/racially diverse sample, preprocessing methods, high-resolution structural brain imaging, and demographic and clinical information of participants (Glasser et al., 2016).

Based on the HCP database, our results indicate volumetric brain differences in white matter structures, subcortical regions, plexus choroids, and total subcortical grey matter between participants identified as African Americans and Whites. Differences in subcortical brain volumetric regions were identified in the bilateral caudate, left thalamus, right globus pallidus, and right ventral diencephalon. Moreover, differences were identified in other brain structures, such as the optic chiasm, the white matter of the right cerebellum, and the corpus callosum in their anterior and posterior portions. In contrast with Isamah et al.’s (2010) study, where differences in bilateral amygdala and total cerebral volume between persons identified as African Americans and White were found, we identified volumetric differences in the bilateral caudate and total cortical white matter. Differences in regional brain volumes in cortical and subcortical structures, such as the bilateral caudate, have been identified between White and Chinese populations (Tang et al., 2010). Moreover, brain differences in total cortical gray matter volume, total cortical white matter volume, total gray matter volume, estimated intracranial volume, and cortical regional volumes have been reported between Indian and White persons (Rao et al., 2017). Furthermore, our results indicate surface area differences in frontal, parietal, temporal, occipital, and frontal brain regions between African American and White racial identities. Specifically, cortical thickness differences were identified in the bilateral cuneus cortex, left fusiform gyrus, bilateral occipital cortex, left pericalcarine cortex, bilateral lingual gyrus, bilateral postcentral gyrus, right superior temporal sulcus, right rostral anterior cingulate cortex, right supramarginal gyrus, right entorhinal cortex, right middle temporal gyrus, and right transverse temporal cortex. Moreover, cortical surface area differences were identified in the bilateral cuneus cortex, left entorhinal cortex, left inferior temporal gyrus, bilateral occipital cortex, left lateral orbitofrontal cortex, left lingual gyrus, bilateral parsopercularis, right parsorbitalis, right caudal middle frontal gyrus, right frontal pole, right fusiform gyrus, bilateral right superior frontal gyrus, and bilateral superior parietal cortex. There are few studies that have reported differences in brain cortical thickness and surface area due to ethnic or racial identity. Accordingly, Jha et al. (2019) identified cortical thickness differences in the bilateral postcentral gyrus, superior parietal lobules, precuneus, supramarginal gyrus, right precentral gyrus, insula, inferior parietal lobule, supplementary motor area, and rolandic operculum in a large cohort of neonates of African American and White mothers. Furthermore, in middle-aged cognitively impaired hypertensive persons, differences in insular cortical thickness were identified between African Americans and White people (Chand et al., 2017). Similar to our results, Kang et al. (2020) identified differences in surface area and cortical thickness in frontal, parietal, temporal, and occipital subregions; however, these results were based on an analysis of brain surface morphometry between older Chinese and White adults.

The U.S. Census has created racial categories that include White and African American people, allowing the self-identification of individuals in groups that represent their community and cultural background (Anderson et al., 2004). In a segregated society, racial identity has emerged as the sense of collective identity based on a perceived common heritage with a racial group (Helms, 1995), promoting wellbeing and protection against racism in African Americans (Hughes et al., 2015). In this sense, Afro-American identity is constituted by an African conscience that establishes behaviors, spirituality, and ancestral knowledge affecting self-concept, self-esteem, and self-image. Moreover, racism and oppression, rooted in a historical background of environmental and interpersonal adversity, have caused a mental and physical pathologization of their identity (Toldson and Toldson, 2001). In contrast, White American identity is rooted in social and economic privileges (McDermott and Samson, 2005) that establish racial attitudes, beliefs, behaviors, and experiences in a racially hierarchical society (Schooley et al., 2019). From this perspective, racial identity is defined and addressed as a social construct from which racial groups are socially created to attach differences between groups (Anderson et al., 2004). In this sense, the descriptive results in our sample related to years of education indicate that participants identified as African American reported less years of education than White participants; moreover, Whites tend to report higher economic income than African Americans. These results may reflect the inequalities in education (Johnson, 2014; Hill et al., 2017) and socioeconomic status (Hardaway and McLoyd, 2008) between White and African American people. Low socioeconomic status has been associated with reduced cortical gray matter thickness in middle-aged persons (Chan et al., 2018). In addition, diverse studies have indicated that socioeconomic status and parental education strongly influence cerebral cortical thickness, surface area, and volume during childhood (Noble et al., 2015; Farah, 2017), particularly average cortical thickness in neonates of African American mothers (Jha et al., 2019). Although our analysis was adjusted for economic income and education, these are only dimensions of socioeconomic status that also imply prenatal and postnatal factors such as biological risks (e.g., nutrition and toxin exposure), psychosocial stress, variability in cognitive and linguistic stimulation, and parenting practices during childhood (Farah, 2017). Our results referred to differences in volume, cortical thickness, and surface area in diverse brain regions between distinct racial identities may be due to these prenatal and postnatal factors anchored in racial inequalities. In this regard, it has been reported that African Americans, compared to the White population, have a higher risk of developing Alzheimer’s disease due to exposure to air pollutants (Younan et al., 2021), access to healthcare (Cooper et al., 2010) and educational disparities (Peterson et al., 2020). Moreover, racism and discrimination have been related to higher levels of blood pressure (Lewis et al., 2009), preterm infant birth (Collins et al., 2011; Dominguez, 2011), and stressful life experiences (Williams, 2018). Furthermore, the recent study by Fani et al. (2021) identified that racial discrimination experiences of Afro-American women were associated with functional activation of the middle occipital gyrus, ventromedial frontal cortex, middle and superior temporal gyrus, and cerebellum. Assari and Mincy (2021) have reported that racism may impact the volume brain growth of African American children. Accordingly, with these studies, the morphological variability identified between White and African American identities in our study may also be related to racism and oppression, mostly affecting the African American community, due to historical racial segregation (Toldson and Toldson, 2001; Grigoryeva and Ruef, 2015). In this regard, acknowledging inequalities in education (Johnson, 2014; Hill et al., 2017), health (Monk, 2015; Yearby, 2018), justice (Hetey and Eberhardt, 2018), and socioeconomic status (Hardaway and McLoyd, 2008) between Whites and African American people is fundamental to acknowledge that racial identity implies social and environmental factors that can impact in human development (Huston and Bentley, 2009) and brain morphology (Ho et al., 2011; Holz et al., 2014; Noble et al., 2015 Gur et al., 2019).

Most studies in human cognitive neuroscience come from majority identities, such as the White population, in contrast to Hispanics, Asians, and African Americans, who have been markedly underrepresented (Dotson et al., 2020). In this sense, our results suggest brain morphological variability between overrepresented and underrepresented samples, supporting the urgency to avoid the extrapolation and generalization of brain findings based on WEIRD (Western, Educated, Industrialized, Rich, and Democratic) population (Chiao and Cheon, 2010; Falk et al., 2013). Accordingly, it is important to consider the human brain as a multilevel ecological system that regards social and biological factors from which it is necessary to develop cross-cultural sampling methods and multidisciplinary collaboration to improve the generalizability of neuroscience studies and the comprehension of individual differences in the human brain (Falk et al., 2013). Neuroimaging research groups have developed structural MRI brain atlas and templates based on specific populations due to differences in brain morphology while contrasting with WEIRD samples (Tang et al., 2010; Gu and Kanai, 2014).

The African Ancestry Neuroscience Research program has emerged as an initiative to reduce health disparities in the African American community and to promote focused brain research in this population to treat brain disorders by developing personalized therapies and treatments (Weinberger et al., 2020). The evidence of morphological brain variability in our study could contribute to understanding brain disorders and psychological factors affecting African Americans and the prospect of developing brain templates for this population.

Although our study was based on a large sample from the HCP database, some limitations must be considered. First, the sample is unbalanced due to the overrepresentation of persons identified as Whites (*n* = 877) compared to persons identified as African Americans (*n* = 193) according to the original HCP database.^2^ Even though the HCP project is focused on neurotypical young adults, this database includes participants with heavy consumption of tobacco, alcohol, and recreational drugs (van Essen et al., 2012). Moreover, we identified participants with psychiatric symptoms, endocrine disorders, irregular menstrual cycles, and neurological abnormalities, as well as technical issues in the acquisition and preprocessing of their structural brain images. In this sense, we consider implementing exclusion criteria to discard these confounding variables that could affect morphological brain results in large neuroimaging data (Smith and Nichols, 2018). Nevertheless, these considerations maintain the imbalance of our sample between Whites (*n* = 338) and African Americans (*n* = 56) persons, which reduces the possibility to apply parametric statistical analysis (Kaur and Kumar, 2015). In this regard, we implement a method of sub-selection of persons identified as White (*n* = 56) and African American (*n* = 56) paired in age, sex, economic income, and education to overcome the confounding bias. Finally, racial identity was defined from the self-identification of participants. However, genetic ancestry information could have contributed to a more careful characterization of the sample from which specific genetic sequences and gene/environmental interactions could be analyzed to further interpret brain morphological results (Fan et al., 2015).

## 5 Conclusion

The human brain is constituted in a unique genetic, social, and experiential domain that is embedded in global hardships such as poverty and discrimination (White and Gonsalves, 2021). In this regard, morphological brain differences in persons identified as African Americans and Whites may be embedded in historical inequalities, oppression, and racism in American society that may impact brain structure. In this study, white matter, forebrain, midbrain, and hindbrain structures display morphological variability between racial groups which could be relevant for understanding neurological or psychiatric disorders differentially affecting these populations. Due to the recurrent misrepresentation of ethnic and racial minorities in neuroimaging research, their inclusion in further studies is fundamental for the comprehension of human brain morphometric variability.

## Data availability statement

Publicly available datasets were analyzed in this study. This data can be found at: https://wiki.humanconnectome.org/display/PublicData/HCP+Wiki+-+Public+Data.

## Ethics statement

The studies involving human participants were reviewed and approved by Data Restricted Access by the Washington University - University of Minnesota Consortium of the Human Connectome Project (WU-Minn HCP). The patients/participants provided their written informed consent to participate in this study.

## Author contributions

DA-B and FB contributed to the conception and design of the study. DA-B organized the database, performed the statistical analysis, and wrote the first draft of the manuscript. FB improved the manuscript by writing additional sections. All authors contributed to manuscript revision, read, and approved the submitted version.

## Footnotes

^1^https://www.humanconnectome.org/storage/app/media/documentation/q3/HCP_Q3_Release_Appendix_VII.pdf

^2^https://wiki.humanconnectome.org/display/PublicData/Summary+Demographic+Data+for+HCP+Young+Adult
