# Supplementary material for: Brain morphological variability between whites and African Americans: the importance of racial identity in brain imaging research
Source: Front Integr Neurosci. 2023 Dec 21;17:1027382. doi: 10.3389/fnint.2023.1027382 (PMC10773238; doi:10.3389/fnint.2023.1027382)
Supplement: Supplementary file 1 [file Data_Sheet_1.DOCX]

***Supplementary Material***

1. **Morphometric brain results due to racial identity in the large HCP sample**

**Table S1**. ANCOVA permutation volumetric brain results between African-Americans and Whites adjusting for *age,sex, education* and *economic income.*

| Volumetric measure  (ICV/mm^3^) | African Americans, N=56  Mean (SD) | Whites, N=355  Mean (SD) | df | MSS | Iteration | p-value | p-adjust value |
| --- | --- | --- | --- | --- | --- | --- | --- |
| Brain Seg (w/o ventricles from surface) | 0.7536 (0.07550) | 0.7350 (0.04062) | 1 | 0.0082 | 5000 | 0.0146 | 0.4964 |
| Brain Seg. | 0.7674 (0.07524) | 0.7478 (0.03997) | 1 | 0.0100 | 1470 | 0.0639 | 1.0000 |
| Brain Seg.  (w/o ventricles) | 0.7548 (0.07559) | 0.7362 (0.04067) | 1 | 0.0083 | 1269 | 0.0732 | 1.0000 |
| Cortical Grey Matter, L | 0.1617 (0.01692) | 0.1599 (0.01084) | 1 | 0.00001 | 51 | 1.0000 | 1.0000 |
| Cortical Grey Matter, R | 0.1653 (0.01732) | 0.1636 (0.01053) | 1 | 0.000007 | 85 | 0.5411 | 1.0000 |
| Total Cortical Grey Matter | 0.3269 (0.03418) | 0.3235 (0.02109) | 1 | 0.00003 | 51 | 0.9803 | 1.0000 |
| Subcortical Grey Matter | 0.04055 (0.005034) | 0.03834 (0.002698) | 1 | 0.00011 | 5000 | 2e-16 | **0.0000***** |
| Total Grey Matter | 0.4407 (0.04499) | 0.4351 (0.02552) | 1 | 0.00013 | 230 | 0.3043 | 1.0000 |
| Supra Tentorial | 0.6726 (0.06674) | 0.6544 (0.03746) | 1 | 0.00909 | 2614 | 0.03711 | 1.0000 |
| Cortical White Matter, L | 0.1456 (0.01530) | 0.1400 (0.01035) | 1 | 0.00112 | 5000 | 0.0004 | **0.0148*** |
| Cortical White Matter, R | 0.148 (0.01591) | 0.142 (0.01041) | 1 | 0.00133 | 5000 | 2e-16 | **0.0000***** |
| Total Cortical White Matter | 0.2936 (0.03118) | 0.2820 (0.02072) | 1 | 0.00491 | 5000 | 2e-16 | **0.0000***** |
| Lateral Ventricle, L | 0.004386 (0.001942) | 0.004069 (0.002128) | 1 | 1.24e-05 | 3021 | 0.03211 | 1.0000 |
| Inferior Lateral Ventricle, L | 0.0001337 (7.621e-05) | 0.0001345 (7.563e-05) | 1 | 1.15e-10 | 51 | 1.000 | 1.0000 |
| Cerebellum White Matter, L | 0.009890 (0.001650) | 0.009156 (0.001009) | 1 | 2.24e-05 | 5000 | 0.0104 | 0.3640 |
| Cerebellar Cortex, L | 0.03627 (0.004179) | 0.03637 (0.003116) | 1 | 1.03e-05 | 1160 | 0.08017 | 1.0000 |
| Thalamus Proper, L | 0.005662 (0.0007313) | 0.005314 (0.0004950) | 1 | 2.97e-06 | 5000 | 2e-16 | **0.0000***** |
| Caudate,L | 0.002561 (0.0003973) | 0.002389 (0.0002699) | 1 | 6.66e-07 | 5000 | 2e-16 | **0.0000***** |
| Putamen, L | 0.003669 (0.0006941) | 0.003470 (0.0004249) | 1 | 7.50e-07 | 2221 | 0.04322 | 1.0000 |
| Pallidum, L | 0.0009094 (0.0002019) | 0.0008439 (0.0001306) | 1 | 1.37e-07 | 594 | 0.1448 | 1.0000 |
| 3rd Ventricle | 0.0004903 (0.0001525) | 0.0004760 (0.0001241) | 1 | 1.62e-08 | 51 | 0.6667 | 1.0000 |
| 4th Ventricle | 0.001153 (0.0004072) | 0.001090 (0.0003352) | 1 | 1.09e-07 | 255 | 0.2823 | 1.0000 |
| Brain Stem | 0.01463 (0.002037) | 0.01375 (0.001411) | 1 | 2.38e-05 | 235 | 0.3021 | 1.0000 |
| Hippocampus, L | 0.002896 (0.0003114) | 0.002798 (0.0002746) | 1 | 7.97e-08 | 51 | 1.0000 | 1.0000 |
| Amygdala, L | 0.0010127 (0.0001142) | 0.0009859 (0.0001046) | 1 | 1.84e-08 | 169 | 0.3728 | 1.0000 |
| Cerebral Spinal Fluid | 0.0006931 (0.0001229) | 0.0006649 (0.0001156) | 1 | 5.74e-08 | 51 | 0.7255 | 1.0000 |
| Accumbens Area, L | 0.0003732 (6.880e-05) | 0.0003560 (5.225e-05) | 1 | 9.36e-09 | 51 | 1.0000 | 1.0000 |
| Ventral Diencephalon, L | 0.002766 (0.0004031) | 0.002658 (0.0002414) | 1 | 2.96e-07 | 95 | 0.5158 | 1.0000 |
| Vessel, L | 4.492e-05 (3.257e-05) | 4.431e-05 (2.552e-05) | 1 | 2.70e-10 | 51 | 1.0000 | 1.0000 |
| Choroid Plexus, L | 0.0007718 (0.0001564) | 0.0006879 (0.0001265) | 1 | 2.74e-07 | 5000 | 2e-16 | **0.0000***** |
| Lateral Ventricle,R | 0.004146 (0.001875) | 0.003753 (0.001902) | 1 | 1.13e-05 | 2866 | 0.03385 | 1.0000 |
| Inferior Lateral Ventricle,R | 0.0001500 (8.713e-05) | 0.0001381 (8.761e-05) | 1 | 7.97e-09 | 51 | 1.0000 | 1.0000 |
| Cerebellum White Matter, R | 0.010071 (0.001618) | 0.009315 (0.001034) | 1 | 2.31e-05 | 5000 | 2e-16 | **0.0000***** |
| Cerebellar Cortex, R | 0.03741 (0.004374) | 0.03739 (0.003251) | 1 | 2.69e-06 | 75 | 0.5733 | 1.0000 |
| Thalamus Proper, R | 0.005033 (0.0006962) | 0.004650 (0.0004254) | 1 | 4.19e-06 | 5000 | 0.0058 | 0.2088 |
| Caudate,R | 0.002643 (0.0003947) | 0.002467 (0.0002746) | 1 | 7.50e-07 | 5000 | 2e-16 | **0.0000***** |
| Putamen, R | 0.003684 (0.0006193) | 0.003502 (0.0003690) | 1 | 5.77e-07 | 2429 | 0.0399 | 1.0000 |
| Pallidum, R | 0.001005 (0.0001865) | 0.000927 (0.0001037) | 1 | 1.76e-07 | 5000 | 2e-16 | **0.0000***** |
| Hippocampus,R | 0.00296 (0.0003008) | 0.00283 (0.0002505) | 1 | 3.58e-07 | 2449 | 0.0396 | 1.0000 |
| Amygdala, R | 0.001073 (0.0001281) | 0.001037 (0.0001166) | 1 | 2.94e-08 | 51 | 1.0000 | 1.0000 |
| Accumbens Area, R | 0.0003955 (6.519e-05) | 0.0003801 (5.413e-05) | 1 | 6.86e-09 | 51 | 1.0000 | 1.0000 |
| Ventral Diencephalon, R | 0.002813 (0.0003966) | 0.002673 (0.0002312) | 1 | 6.68e-07 | 5000 | 2e-16 | **0.0000***** |
| Vessel,R | 5.08e-05 (3.158e-05) | 4.94e-05 (2.359e-05) | 1 | 2.33e-11 | 51 | 1.0000 | 1.0000 |
| Choroid Plexus,R | 0.0008843 (0.0002290) | 0.0007852 (0.0001559) | 1 | 4.19e-07 | 5000 | 2e-16 | **0.0000***** |
| 5th Ventricle | 2.376e-06 (3.645e-06) | 2.553e-06 (3.227e-06) | 1 | 2.73e-13 | 51 | 1.0000 | 1.0000 |
| Optic Chiasm | 0.0001660 (3.156e-05) | 0.0001423 (2.936e-05) | 1 | 2.83e-08 | 5000 | 2e-16 | **0.0000***** |
| Posterior Corpus Callosum | 0.0006727 (1.129e-04) | 0.0005998 (9.114e-05) | 1 | 1.44e-07 | 5000 | 2e-16 | **0.0000***** |
| Mid Posterior Corpus Callosum | 0.0003024 (7.683e-05) | 0.0002950 (6.533e-05) | 1 | 3.38e-10 | 51 | 1.0000 | 1.0000 |
| Central Corpus Callosum | 0.0003310 (6.916e-05) | 0.0003151 (7.108e-05) | 1 | 2.93e-09 | 51 | 1.0000 | 1.0000 |
| Mid Anterior Corpus Callosum | 0.0003322 (7.376e-05) | 0.0003113 (6.725e-05) | 1 | 8.13e-09 | 51 | 1.0000 | 1.0000 |
| Anterior Corpus Callosum | 0.0006323 (9.232e-05) | 0.0005577 (8.614e-05) | 1 | 1.75e-07 | 5000 | 2e-16 | **0.0000***** |
| *Note*: (***) Significant results at p < .001**,** and (*) p < .05 when multiple comparison correction test (FWER) was applied. MSS: Mean sum of squares | | | | | | | |

**Table S2**. Permutational ANCOVA cortical thickness results between African-Americans and Whites adjusting for *age, sex, education* and *economic income.*

| Brain  Region  (mm) | African Americans, N=56  Mean (SD) | Whites,  N=355  Mean (SD) | df | MSS | Iteration | p-value | p-adjust value |
| --- | --- | --- | --- | --- | --- | --- | --- |
| Banks of superior temporal sulcus, L | 2.652 (0.1345) | 2.693 (0.1414) | 1 | 0.07713 | 5000 | 0.01260 | 0.6174 |
| Banks of superior temporal sulcus, R | 2.744 (0.1603) | 2.810 (0.1342) | 1 | 0.21868 | 5000 | 2e-16 | **0.0000***** |
| Caudal Anterior Cingulate Cortex, L | 2.742 (0.1818) | 2.687 (0.1650) | 1 | 0.09188 | 51 | 0.96078 | 1.0000 |
| Caudal Anterior Cingulate Cortex, R | 2.536 (0.2326) | 2.504 (0.2369) | 1 | 0.01143 | 51 | 0.9411 | 1.0000 |
| Caudal Middle Frontal Gyrus, L | 2.762 (0.126) | 2.728 (0.126) | 1 | 0.05319 | 776 | 0.1147 | 1.0000 |
| Caudal Middle Frontal Gyrus, R | 2.777 (0.1229) | 2.742 (0.1170) | 1 | 0.06656 | 5000 | 0.0084 | 0.4200 |
| Cuneus Cortex, L | 2.040 (0.1011) | 2.099 (0.1214) | 1 | 0.20477 | 5000 | 2e-16 | **0.0000***** |
| Cuneus Cortex, R | 2.042 (0.1147) | 2.099 (0.1105) | 1 | 0.17220 | 5000 | 2e-16 | **0.0000***** |
| Entorhinal Cortex, L | 3.271 (0.2034) | 3.318 (0.2490) | 1 | 0.07503 | 925 | 0.09838 | 1.0000 |
| Entorhinal Cortex, R | 3.313 (0.2290) | 3.445 (0.2412) | 1 | 0.64413 | 5000 | 2e-16 | **0.0000***** |
| Frontal Pole, L | 2.870 (0.2009) | 2.825 (0.2009) | 1 | 0.0864 | 454 | 0.1806 | 1.0000 |
| Frontal Pole, R | 2.901 (0.2236) | 2.854 (0.2021) | 1 | 0.0960 | 805 | 0.1106 | 1.0000 |
| Fusiform Gyrus, L | 2.850 (0.09736) | 2.889 (0.12094) | 1 | 0.06748 | 5000 | 2e-16 | **0.0000***** |
| Fusiform Gyrus, R | 2.860 (0.1161) | 2.906 (0.1104) | 1 | 0.09658 | 1997 | 0.04807 | 1.0000 |
| Inferior Parietal Cortex, L | 2.552 (0.1017) | 2.585 (0.1118) | 1 | 0.06394 | 5000 | 0.0072 | 0.3672 |
| Inferior Parietal Cortex, R | 2.618 (0.09668) | 2.657 (0.10264) | 1 | 0.09692 | 5000 | 2e-16 | **0.0000***** |
| Inferior Temporal Gyrus, L | 2.930 (0.1036) | 2.954 (0.1289) | 1 | 0.02431 | 81 | 0.55556 | 1.0000 |
| Inferior Temporal Gyrus, R | 2.954 (0.1147) | 2.991 (0.1221) | 1 | 0.05500 | 1719 | 0.05526 | 1.0000 |
| Insula, L | 3.020 (0.1444) | 3.067 (0.1443) | 1 | 0.03898 | 997 | 0.09127 | 1.0000 |
| Insula, R | 2.969  (0.1447) | 3.025 (0.1455) | 1 | 0.06265 | 51 | 1.0000 | 1.0000 |
| Isthmus of Cingulate Gyrus, L | 2.273 (0.2116) | 2.290 (0.2969) | 1 | 0.06540 | 231 | 0.3030 | 1.0000 |
| Isthmus of Cingulate Gyrus, R | 2.304 (0.1802) | 2.336 (0.1715) | 1 | 0.03067 | 1054 | 0.08729 | 1.0000 |
| Lateral Occipital Cortex, L | 2.234 (0.1040) | 2.312 (0.1099 | 1 | 0.27342 | 5000 | 2e-16 | **0.0000***** |
| Lateral Occipital Cortex, R | 2.273 (0.1102) | 2.366 (0.1036) | 1 | 0.35269 | 5000 | 2e-16 | **0.0000***** |
| Lateral Orbitofrontal Cortex, L | 2.789 (0.1476) | 2.817 (0.1265) | 1 | 0.02047 | 468 | 0.17735 | 1.0000 |
| Lateral Orbitofrontal Cortex, R | 2.808 (0.1417) | 2.844 (0.1278)0.06265 | 1 | 0.03526 | 1448 | 0.06492 | 1.0000 |
| Lingual Gyrus, L | 2.125 (0.09406) | 2.189 (0.11238) | 1 | 0.14523 | 5000 | 2e-16 | **0.0000***** |
| Lingual Gyrus, R | 2.132 (0.1075) | 2.208 (0.1101) | 1 | 0.23351 | 5000 | 2e-16 | **0.0000***** |
| Medial Orbitofrontal Cortex, L | 2.619 (0.1388) | 2.555 (0.1610) | 1 | 0.16711 | 2994 | 0.0324 | 1.0000 |
| Medial Orbitofrontal Cortex, R | 2.774 (0.1516) | 2.737 (0.1398) | 1 | 0.06265 | 5000 | 0.0186 | 0.8370 |
| Middle Temporal Gyrus, L | 2.950 (0.1184) | 2.991 (0.1347) | 1 | 0.06880 | 1248 | 0.07452 | 1.0000 |
| Middle Temporal Gyrus, R | 3.013 (0.1111) | 3.074 (0.1211) | 1 | 0.13934 | 5000 | 2e-16 | **0.0000***** |
| Paracentral Sulcus, L | 2.542 (0.1193) | 2.569 (0.1365) | 1 | 0.02775 | 51 | 1.0000 | 1.0000 |
| Paracentral Sulcus, R | 2.588 (0.1187) | 2.595 (0.1307) | 1 | 0.00661 | 179 | 0.3631 | 1.0000 |
| Parahippocampal Gyrus, L | 2.707 (0.2213) | 2.730 (0.2678) | 1 | 0.08209 | 53 | 0.6604 | 1.0000 |
| Parahippocampal Gyrus, R | 2.656 (0.1573) | 2.712 (0.2310) | 1 | 0.17544 | 5000 | 0.0174 | 0.8178 |
| Parsopercularis, L | 2.796 (0.1117) | 2.778 (0.1306) | 1 | 0.01877 | 319 | 0.2414 | 1.0000 |
| Parsopercularis, R | 2.854 (0.1314) | 2.827 (0.1131) | 1 | 0.02952 | 531 | 0.1601 | 1.0000 |
| Parsorbitalis, L | 2.790 (0.1663) | 2.777 (0.1536) | 1 | 0.00261 | 51 | 1.00000 | 1.0000 |
| Parsorbitalis, R | 2.843 (0.1390) | 2.843 (0.1509) | 1 | 0.000002 | 51 | 1.0000 | 1.0000 |
| Parstriangularis, L | 2.634 (0.1437) | 2.629 (0.1285) | 1 | 0.00155 | 51 | 1.0000 | 1.0000 |
| Parstriangularis, R | 2.703 (0.1381) | 2.697 (0.1223) | 1 | 0.00040 | 51 | 1.0000 | 1.0000 |
| Pericalcarine Cortex, L | 1.978 (0.1141) | 2.020 (0.1170) | 1 | 0.11471 | 5000 | 2e-16 | **0.0000***** |
| Pericalcarine Cortex, R | 1.982 (0.1258) | 2.020 (0.1122) | 1 | 0.05218 | 2462 | 0.0394 | 1.0000 |
| Postcentral Gyrus, L | 2.176 (0.09014) | 2.230 (0.10179) | 1 | 0.12848 | 5000 | 2e-16 | **0.0000***** |
| Postcentral Gyrus, R | 2.204 (0.10093) | 2.248 (0.09787) | 1 | 0.08111 | 5000 | 2e-16 | **0.0000***** |
| Posterior Cingulate Gyrus, L | 2.582 (0.1247) | 2.564 (0.1298) | 1 | 0.00645 | 309 | 0.2460 | 1.0000 |
| Posterior Cingulate Gyrus, R | 2.511 (0.1506) | 2.532 (0.1564) | 1 | 0.02699 | 297 | 0.25253 | 1.0000 |
| Precentral Gyrus, L | 2.740 (0.09945) | 2.738 (0.12175) | 1 | 0.00009 | 51 | 1.00000 | 1.0000 |
| Precentral Gyrus, R | 2.739 (0.1121) | 2.739 (0.1122) | 1 | 0.00047 | 51 | 1.0000 | 1.0000 |
| Precuneus Cortex, L | 2.514 (0.1153) | 2.533 (0.1143) | 1 | 0.01611 | 1159 | 0.08024 | 1.0000 |
| Precuneus Cortex, R | 2.543 (0.1150) | 2.566 (0.1096) | 1 | 0.02758 | 2677 | 0.03623 | 1.0000 |
| Rostral Anterior Cingulate Cortex, L | 3.081 (0.1964) | 3.020 (0.1954) | 1 | 0.19269 | 5000 | 0.0138 | 1.0000 |
| Rostral Anterior Cingulate Cortex, R | 3.056 (0.1737) | 2.996 (0.1853) | 1 | 0.18686 | 5000 | 0.0006 | **0.0312*** |
| Rostral Middle Frontal Gyrus, L | 2.584 (0.1360) | 2.567 (0.1193) | 1 | 0.02097 | 83 | 0.5542 | 1.0000 |
| Rostral Middle Frontal Gyrus, R | 2.600 (0.1323) | 2.588 (0.1126) | 1 | 0.01565 | 349 | 0.2235 | 1.0000 |
| Superior Frontal Gyrus, L | 2.869 (0.1321) | 2.832 (0.1364) | 1 | 0.06199 | 603 | 0.1426 | 1.0000 |
| Superior Frontal Gyrus, R | 2.897 (0.1461) | 2.869 (0.1276) | 1 | 0.02795 | 99 | 0.5051 | 1.0000 |
| Superior Parietal Cortex, L | 2.281 (0.1039) | 2.294 (0.1055) | 1 | 0.01254 | 430 | 0.1907 | 1.0000 |
| Superior Parietal Cortex, R | 2.316 (0.1015) | 2.333 (0.1023) | 1 | 0.02213 | 953 | 0.0954 | 1.0000 |
| Superior Temporal Gyrus, L | 2.888 (0.1137) | 2.902 (0.1368) | 1 | 0.00192 | 154 | 0.3961 | 1.0000 |
| Superior Temporal Gyrus, R | 2.942 (0.1237) | 2.958 (0.1265) | 1 | 0.00011 | 51 | 1.0000 | 1.0000 |
| Supramarginal Gyrus, L | 2.633 (0.1116) | 2.665 (0.1162) | 1 | 0.06518 | 3703 | 0.02647 | 1.0000 |
| Supramarginal Gyrus, R | 2.668 (0.1017) | 2.715 (0.1152) | 1 | 0.14505 | 5000 | 2e-16 | **0.0000***** |
| Temporal Pole, L | 3.411 (0.2510) | 3.420 (0.2569) | 1 | 0.0046 | 51 | 1.0000 | 1.0000 |
| Temporal Pole, R | 3.588 (0.2633) | 3.671 (0.2895) | 1 | 0.2362 | 813 | 0.1107 | 1.0000 |
| Transverse Temporal Cortex, L | 2.598 (0.1683) | 2.667 (0.1741) | 1 | 0.22038 | 5000 | 0.01780 | 0.8188 |
| Transverse Temporal Cortex, R | 2.653 (0.1645) | 2.765 (0.1709) | 1 | 0.50098 | 5000 | 2e-16 | **0.0000***** |
| *Note*: (***) Significant results at p < .001 **,** and (*) p < .05 when multiple comparison correction test (FWER) was applied. MSS: Mean sum of squares | | | | | | | |

**Table S3.** Permutational ANCOVA surface cortical area results between African-Americans and Whites adjusting for *age, sex, education* and *economic income.*

| Brain  Region  (mm^2^) | African Americans, N=56  Mean (SD) | Whites,  N=355  Mean (SD) | df | MSS | Iteration | p-value | p-adjust value |
| --- | --- | --- | --- | --- | --- | --- | --- |
| Banks of superior temporal sulcus, L | 1053.95 (181.688) | 1090.33  (187.857) | 1 | 2242 | 51 | 0.68627 | 1.0000 |
| Banks of superior temporal sulcus, R | 951.625 (144.698) | 1009.158 (158.446) | 1 | 43679 | 51 | 0.94118 | 1.0000 |
| Caudal Anterior Cingulate Cortex, L | 691.214 (171.189) | 704.609 (142.164) | 1 | 13392 | 414 | 0.19565 | 1.0000 |
| Caudal Anterior Cingulate Cortex, R | 790.232 (214.645) | 852.931 (224.018) | 1 | 43447 | 212 | 0.3208 | 1.0000 |
| Caudal Middle Frontal Gyrus, L | 2227.34 (411.954) | 2449.64 (419.162) | 1 | 844556 | 5000 | 0.00600 | 0.2760 |
| Caudal Middle Frontal Gyrus, R | 2031.68 (430.703) | 2289.21 (412.912) | 1 | 1313656 | 5000 | 2e-16 | **0.0000***** |
| Cuneus Cortex, L | 1379.70 (213.901) | 1506.87 (219.553) | 1 | 279408 | 5000 | 0.0186 | 0.7440 |
| Cuneus Cortex, R | 1404.12 (203.642) | 1544.09 (240.150) | 1 | 468061 | 5000 | 0.0094 | 0.4136 |
| Entorhinal Cortex, L | 392.036 (79.9331) | 442.269 (89.4814) | 1 | 58967 | 5000 | 2e-16 | **0.0000***** |
| Entorhinal Cortex, R | 340.768 (67.1617) | 371.988 (85.5521) | 1 | 13164 | 81 | 0.5556 | 1.00000 |
| Frontal Pole, L | 195.089 (33.9761) | 208.149 (33.7204) | 1 | 2227 | 2690 | 0.0360 | 1.0000 |
| Frontal Pole, R | 255.232 (41.2782) | 286.684 (46.4144) | 1 | 22534 | 5000 | 2e-16 | **0.0000***** |
| Fusiform Gyrus, L | 3241.14 (494.378) | 3462.79  (460.202) | 1 | 353020 | 2326 | 0.04127 | 1.0000 |
| Fusiform Gyrus, R | 3091.07 (409.400) | 3385.21 (485.103) | 1 | 979123 | 5000 | 0.0004 | **0.0208*** |
| Inferior Parietal Cortex, L | 4555.73 (697.133) | 4784.13 (697.784) | 1 | 132018 | 72 | 0.5833 | 1.0000 |
| Inferior Parietal Cortex, R | 5466.62 (756.464) | 5770.69 (837.977) | 1 | 266772 | 51 | 1.0000 | 1.0000 |
| Inferior Temporal Gyrus, L | 3151.93 (459.970) | 3535.21 (514.899) | 1 | 2869915 | 5000 | 2e-16 | **0.0000***** |
| Inferior Temporal Gyrus, R | 3074.48 (461.852) | 3392.64 (493.629) | 1 | 1701978 | 5000 | 0.0040 | 0.1960 |
| Insula, L | 2184.98 (256.330) | 2335.29 (290.999) | 1 | 226417 | 2462 | 0.0394 | 1.0000 |
| Insula, R | 2321.86 (310.777) | 2482.65 (296.405) | 1 | 195775 | 1139 | 0.0807 | 1.0000 |
| Isthmus of Cingulate Gyrus, L | 1039.59 (396.803) | 1146.46 (367.334) | 1 | 79941 | 191 | 0.3455 | 1.0000 |
| Isthmus of Cingulate Gyrus, R | 947.804 (158.111) | 1004.970 (185.272) | 1 | 7557 | 51 | 1.0000 | 1.0000 |
| Lateral Occipital Cortex, L | 4432.77 (606.078) | 4869.55  (621.979) | 1 | 4366717 | 5000 | 2e-16 | **0.0000***** |
| Lateral Occipital Cortex, R | 4281.59 (629.353) | 4734.67 (607.326) | 1 | 3922367 | 5000 | 2e-16 | **0.0000***** |
| Lateral Orbitofrontal Cortex, L | 2511.55 (288.722) | 2733.41 (318.287) | 1 | 703296 | 5000 | 2e-16 | **0.0000***** |
| Lateral Orbitofrontal Cortex, R | 2486.0 (289.365) | 2657.8 (320.907) | 1 | 311889 | 5000 | 0.01080 | 0.4644 |
| Lingual Gyrus, L | 2863.04 (396.938) | 3190.75 (415.092) | 1 | 2569762 | 5000 | 2e-16 | **0.0000***** |
| Lingual Gyrus, R | 2956.55 (423.849) | 3199.50 (422.641) | 1 | 919325 | 5000 | 0.00400 | 0.1960 |
| Medial Orbitofrontal Cortex, L | 1927.62 (329.580) | 2040.33 (310.594) | 1 | 67876 | 371 | 0.2129 | 1.0000 |
| Medial Orbitofrontal Cortex, R | 1773.02 (219.818) | 1900.18 (240.230) | 1 | 180122 | 5000 | 0.0150 | 0.6150 |
| Middle Temporal Gyrus, L | 2960.05 (441.082) | 3255.31  (459.286) | 1 | 1452636 | 5000 | 0.0028 | 0.1400 |
| Middle Temporal Gyrus, R | 3306.09 (413.054) | 3617.42 (485.336) | 1 | 1727105 | 5000 | 0.0010 | 0.0510 |
| Paracentral Sulcus, L | 1313.38 (164.918) | 1341.30 (200.675) | 1 | 12605 | 304 | 0.2500 | 1.0000 |
| Paracentral Sulcus, R | 1520.82 (249.460) | 1532.75 (226.837) | 1 | 28543 | 51 | 0.7451 | 1.0000 |
| Parahippocampal Gyrus, L | 718.964 (117.083) | 752.893  (128.354) | 1 | 652 | 51 | 0.8039 | 1.0000 |
| Parahippocampal Gyrus, R | 685.071 ( 90.1848) | 720.934 (114.3897) | 1 | 2722 | 219 | 0.3151 | 1.0000 |
| Parsopercularis, L | 1589.21 (246.493) | 1777.46 (293.377) | 1 | 774206 | 5000 | 2e-16 | **0.0000***** |
| Parsopercularis, R | 1292.12 (226.984) | 1498.99  (265.773) | 1 | 1301307 | 5000 | 2e-16 | **0.0000***** |
| Parsorbitalis, L | 613.179 (78.1756) | 660.054 (84.4324) | 1 | 26448 | 1263 | 0.07363 | 1.0000 |
| Parsorbitalis, R | 745.054 ( 95.406) | 822.463  (106.355) | 1 | 90705 | 5000 | 2e-16 | **0.0000***** |
| Parstriangularis, L | 1273.68 (212.375) | 1338.01 (216.540) | 1 | 10705 | 160 | 0.3875 | 1.0000 |
| Parstriangularis, R | 1520.66 (239.653) | 1542.83 (258.475) | 1 | 20173 | 51 | 0.94118 | 1.0000 |
| Pericalcarine Cortex, L | 1385.70 (264.149) | 1511.64 (248.123) | 1 | 348897 | 5000 | 0.0186 | 0.7440 |
| Pericalcarine Cortex, R | 1550.45 (263.249) | 1641.83 (254.456) | 1 | 142518 | 699 | 0.1259 | 1.0000 |
| Postcentral Gyrus, L | 3998.84 (517.208) | 4240.89  (514.257) | 1 | 379736 | 339 | 0.2300 | 1.0000 |
| Postcentral Gyrus, R | 3881.04 (535.196) | 4045.89  (484.084) | 1 | 52923 | 130 | 0.4384 | 1.0000 |
| Posterior Cingulate Gyrus, L | 1201.57 (152.927) | 11241.95 (193.186) | 1 | 2390 | 51 | 1.0000 | 1.0000 |
| Posterior Cingulate Gyrus, R | 1187.12 (169.303) | 1301.06 (235.390) | 1 | 168394 | 3828 | 0.02560 | 0.9728 |
| Precentral Gyrus, L | 4599.20 (549.386) | 4928.76 (618.145) | 1 | 1391310 | 1001 | 0.09091 | 1.0000 |
| Precentral Gyrus, R | 4691.07 (549.048) | 5014.66  (590.347) | 1 | 1116311 | 766 | 0.11619 | 1.0000 |
| Precuneus Cortex, L | 3610.70 (519.087) | 3951.11 (511.976) | 1 | 1716490 | 5000 | 0.0056 | 0.2632 |
| Precuneus Cortex, R | 3770.21 (527.036) | 4201.71  (609.588) | 1 | 2710670 | 5000 | 2e-16 | **0.0000***** |
| Rostral Anterior Cingulate Cortex, L | 830.250 (159.754) | 903.773 (17122534.607) | 1 | 31124 | 809 | 0.11001 | 1.0000 |
| Rostral Anterior Cingulate Cortex, R | 630.768 (148.744) | 698.143 (142.306) | 1 | 60154 | 5000 | 0.0134 | 0.5628 |
| Rostral Middle Frontal Gyrus, L | 5810.46 (796.104) | 6178.43 (844.455) | 1 | 864595 | 51 | 1.0000 | 1.0000 |
| Rostral Middle Frontal Gyrus, R | 6039.79 (836.828) | 6414.55 (883.613) | 1 | 694697 | 843 | 0.1067 | 1.0000 |
| Superior Frontal Gyrus, L | 6931.23 (868.670) | 7610.22 (962.951) | 1 | 7349511 | 5000 | 2e-16 | **0.0000***** |
| Superior Frontal Gyrus, R | 6849.00 (828.756) | 7445.82 (928.692) | 1 | 4132493 | 5000 | 2e-16 | **0.0000***** |
| Superior Parietal Cortex, L | 5080.77 (565.338) | 5680.33 (731.003) | 1 | 6302632 | 5000 | 2e-16 | **0.0000***** |
| Superior Parietal Cortex, R | 5100.62 (558.739) | 5704.45 (699.319) | 1 | 6535061 | 5000 | 2e-16 | **0.0000***** |
| Superior Temporal Gyrus, L | 3760.05 (455.162) | 3946.04 (491.293) | 1 | 29330 | 124 | 0.45161 | 1.0000 |
| Superior Temporal Gyrus, R | 3600.50 (413.430) | 3738.62 (442.197) | 1 | 139 | 51 | 1.0000 | 1.0000 |
| Supramarginal Gyrus, L | 3767.02 (554.727) | 4050.87 (631.802) | 1 | 346376 | 942 | 0.0966 | 1.0000 |
| Supramarginal Gyrus, R | 3617.39 (589.364) | 3837.79 (572.235) | 1 | 190134 | 239 | 0.2970 | 1.0000 |
| Temporal Pole, L | 477.411 (60.3501) | 508.263 (61.7090) | 1 | 15398 | 5000 | 0.0082 | 0.3690 |
| Temporal Pole, R | 436.429 (69.3919) | 437.331  (57.5140) | 1 | 5759 | 995 | 0.0914 | 1.0000 |
| Transverse Temporal Cortex, L | 445.768 (76.5589) | 464.734 (82.6921) | 1 | 359 | 143 | 0.4126 | 1.0000 |
| Transverse Temporal Cortex, R | 332.071 (58.5454) | 344.227 (60.3191) | 1 | 191 | 51 | 0.7254 | 1.0000 |
| *Note*: (***) Significant results at p < .001, and (*) p < .05 when multiple comparison correction test (FWER) was applied. MSS: Mean sum of squares | | | | | | | |

1. **Descriptive results due to racial identity in the paired HCP sample**

**Table S4.** Descriptive results between African-Americans and Whites of the paired subsample.

|  | **N** | Racial Identity | | **p-value***^2^* |
| --- | --- | --- | --- | --- |
|  |  | **African-Americans**,  N = 56*^1^* | **Whites**,  N =56 *^1^* |  |
| **Age (y)** | 112 | 29.25 (3.62) | 29.18 (3.79) | 0.919 |
| **Education(y)** |  | 14.41 (1.90) | 14.27 (1.80) | 0.684 |
| **Sex** |  |  |  | >0.999 |
| Female |  | 33 (59%) | 33 (59%) |  |
| Male |  | 23 (41%) | 23 (41%) |  |
| **Economic Income** | 112 |  |  | 0.527 |
| <$10000 |  | 7 (12%) | 3(5.4%) |  |
| 10K-19999 |  | 10 (18%) | 5 (8.9%) |  |
| 20K-29999 |  | 9 (16%) | 9 (16%) |  |
| 30K-39999 |  | 10 (18%) | 8(14%) |  |
| 40K-49999 |  | 5 (8.9%) | 9 (16%) |  |
| 50K-74999 |  | 8 (14%) | 13 (23%) |  |
| 75K-99999 |  | 1 (1.8%) | 2 (3.6%) |  |
| >=100000 |  | 6 (11%) | 7 (12%) |  |
| ^1^ Mean (SD) of age and education in years. Frequencies (n) and percentages (%) of economic income ranges in US dollars.^2^ Welch Two Sample t-test of age and education between African Americans and Whites (p < .05). Pearson's Chi-squared test of sex frequencies between African Americans and Whites (p < .05). Fisher's exact test of economic income between African Americans and Whites (p < .05). | | | | |

1. **Morphometric brain results due to racial identity in the paired HCP sample.**

**Table S5.** ANCOVA permutation volumetric brain results between African-Americans and Whites adjusting for *age,sex, education* and *economic income* from the paired subsample.

| Volumetric Measure  (ICV/mm^3^) | **African-Americans**  N = 56 | **Whites**  N = 56 | df | MSS | Iteration | p-value | p-adjust value |
| --- | --- | --- | --- | --- | --- | --- | --- |
| Brain Seg. | 0.77 (0.075) | 0.74 (0.025) | 1 | 0.01812 | 4047 | **0.0242*** | 0.8475 |
| Brain Seg.  (w/o ventricles) | 0.75 (0.076) | 0.73 (0.027) | 1 | 0.01687 | 3375 | **0.0290*** | 0.9291 |
| Brain Seg (w/o ventricles from surface) | 0.75 (0.075) | 0.73 (0.027) | 1 | 0.01677 | 3916 | **0.0250*** | 0.8475 |
| Cortical Grey Matter, L | 0.16 (0.0169) | 0.16 (0.0094) | 1 | 0.00025 | 1262 | 0.0736 | 1.0000 |
| Cortical Grey Matter, R | 0.17 (0.0173) | 0.16 (0.0074) | 1 | 0.00016 | 51 | 0.8431 | 1.0000 |
| Total Cortical Grey Matter | 0.33 (0.034) | 0.32 (0.016) | 1 | 0.00084 | 138 | 0.4203 | 1.0000 |
| Subcortical Grey Matter | 0.041 (0.0050) | 0.038 (0.0025) | 1 | 9.305e-16 | 5000 | **0.0170*** | 0.6120 |
| Total Grey Matter | 0.44 (0.045) | 0.43 (0.019) | 1 | 0.00127 | 531 | 0.1601 | 1.0000 |
| Supra Tentorial | 0.67 (0.067) | 0.65 (0.026) | 1 | 0.01712 | 5000 | **0.0110*** | 0.4290 |
| Cortical White Matter, L | 0.15 (0.0153) | 0.14 (0.0078) | 1 | 0.00180 | 5000 | **0.0006***** | **0.0282*** |
| Cortical White Matter, R | 0.15 (0.0159) | 0.14 (0.0081) | 1 | 0.00201 | 5000 | **0.0002***** | **0.0098**** |
| Total Cortical White Matter | 0.29 (0.031) | 0.28 (0.016) | 1 | 0.00763 | 5000 | **0.0006***** | **0.0282*** |
| Lateral Ventricle, L | 0.0044 (0.0019) | 0.0040 (0.0024) | 1 | 1.910e-06 | 51 | 0.9804 | 1.0000 |
| Inferior Lateral Ventricle, L | 0.00013 (7.6e-05) | 0.00013 (6.9e-05) | 1 | 108.84 | 51 | 1.0000 | 1.0000 |
| Cerebellum White Matter, L | 0.0099 (0.00165) | 0.0092 (0.00089) | 1 | 1.311e-05 | 5000 | **0.0010***** | **0.0450*** |
| Cerebellar Cortex, L | 0.036 (0.0042) | 0.036 (0.0032) | 1 | 2.559e-06 | 53 | 0.6603 | 1.0000 |
| Thalamus Proper, L | 0.0057 (0.00073) | 0.0053 (0.00042) | 1 | 3.835e-06 | 5000 | **2e-16***** | **0.0000***** |
| Caudate,L | 0.0026 (0.00040) | 0.0024 (0.00024) | 1 | 4.241e-07 | 5000 | **0.0084**** | 0.3444 |
| Putamen, L | 0.0037 (0.00069) | 0.0035 (0.00043) | 1 | 4.363e-07 | 238 | 0.2983 | 1.0000 |
| Pallidum, L | 0.00091 (0.00020) | 0.00084 (0.00013) | 1 | 5.833e-08 | 51 | 0.7451 | 1.0000 |
| 3rd Ventricle | 0.00049 (0.00015) | 0.00048 (0.00014) | 1 | 3.562e-10 | 51 | 1.0000 | 1.0000 |
| 4th Ventricle | 0.0012 (0.00041) | 0.0011 (0.00032) | 1 | 3.682e-08 | 121 | 0.4545 | 1.0000 |
| Brain Stem | 0.015 (0.00204) | 0.014 (0.00091) | 1 | 1.994e-05 | 5000 | **0.0002***** | **0.0098**** |
| Hippocampus, L | 0.0029 (0.00031) | 0.0028 (0.00022) | 1 | 8.965e-08 | 51 | 0.8823 | 1.0000 |
| Amygdala, L | 0.00101 (1.1e-04) | 0.00099 (9.4e-05) | 1 | 1.836e-08 | 51 | 0.9608 | 1.0000 |
| Cerebral Spinal Fluid | 0.00069 (0.00012) | 0.00065 (0.00012) | 1 | 4.057e-08 | 1250 | 0.0744 | 1.0000 |
| Accumbens Area, L | 0.00037 (6.9e-05) | 0.00034 (5.0e-05) | 1 | 1.095e-08 | 150 | 0.4000 | 1.0000 |
| Ventral Diencephalon, L | 0.0028 (4e-04) | 0.0027 (2e-04) | 1 | 1.782e-07 | 51 | 0.8235 | 1.0000 |
| Vessel, L | 4.5e-05 (3.3e-05) | 4.6e-05 (3.0e-05) | 1 | 4.537e-11 | 51 | 1.0000 | 1.0000 |
| Choroid Plexus, L | 0.00077 (0.00016) | 0.00069 (0.00012) | 1 | 1.4001e-07 | 5000 | **0.0088**** | 0.3520 |
| Lateral Ventricle,R | 0.0041 (0.0019) | 0.0036 (0.0019) | 1 | 4.373e-06 | 198 | 0.3384 | 1.0000 |
| Inferior Lateral Ventricle,R | 0.00015 (8.7e-05) | 0.00014 (8.0e-05) | 1 | 9.052e-09 | 51 | 1.0000 | 1.0000 |
| Cerebellum White Matter, R | 0.0101 (0.00162) | 0.0094 (0.00094) | 1 | 1.083e-05 | 5000 | **0.0046**** | 0.1978 |
| Cerebellar Cortex, R | 0.037 (0.0044) | 0.037 (0.0033) | 1 | 1.486e-06 | 51 | 1.0000 | 1.0000 |
| Thalamus Proper, R | 0.0050 (0.00070) | 0.0046 (0.00039) | 1 | 3.611e-06 | 5000 | **2e-16***** | **0.0000***** |
| Caudate,R | 0.0026 (0.00039) | 0.0025 (0.00025) | 1 | 4.331e-07 | 1809 | 0.0525 | 1.0000 |
| Putamen, R | 0.0037 (0.00062) | 0.0035 (0.00039) | 1 | 8.277e-07 | 1404 | 0.0669 | 1.0000 |
| Pallidum, R | 0.00100 (0.00019) | 0.00092 (0.00011) | 1 | 1.157e-07 | 2461 | **0.0394*** | 1.0000 |
| Hippocampus,R | 0.0030 (0.00030) | 0.0029 (0.00021) | 1 | 1.885e-07 | 1555 | 0.0604 | 1.0000 |
| Amygdala, R | 0.0011 (1.3e-04) | 0.0010 (9.5e-05) | 1 | 4.105e-08 | 729 | 0.1207 | 1.0000 |
| Accumbens Area, R | 0.00040 (6.5e-05) | 0.00037 (5.3e-05) | 1 | 8.663e-09 | 51 | 1.0000 | 1.0000 |
| Ventral Diencephalon, R | 0.0028 (0.00040) | 0.0027 (0.00022) | 1 | 4.020e-09 | 5000 | **0.0148*** | 0.5476 |
| Vessel,R | 5.1e-05 (3.2e-05) | 5.3e-05 (3.0e-05) | 1 | 3.639e-10 | 51 | 1.0000 | 1.0000 |
| Choroid Plexus,R | 0.00088 (0.00023) | 0.00078 (0.00013) | 1 | 2.090e-07 | 5000 | **0.0110*** | 0.4290 |
| 5th Ventricle | 2.4e-06 (3.6e-06) | 3.0e-06 (3.7e-06) | 1 | 1.230e-11 | 51 | 1.0000 | 1.0000 |
| Optic Chiasm | 0.00017 (3.2e-05) | 0.00014 (3.0e-05) | 1 | 1.436e-08 | 3937 | **0.0248*** | 0.8475 |
| Posterior Corpus Callosum^3^ | 0.00067 (1.1e-04) | 0.00061 (9.5e-05) | 1 | 9.834e-08 | 5000 | **0.0046**** | 0.1978 |
| Mid Posterior Corpus Callosum | 0.00030 (7.7e-05) | 0.00029 (7.7e-05) | 1 | 1.811e-09 | 51 | 1.0000 | 1.0000 |
| Central Corpus Callosum | 0.00033 (6.9e-05) | 0.00033 (8.0e-05) | 1 | 1.408e-09 | 51 | 1.0000 | 1.0000 |
| Mid Anterior Corpus Callosum | 0.00033 (7.4e-05) | 0.00032 (7.8e-05) | 1 | 2.748e-09 | 51 | 1.0000 | 1.0000 |
| Anterior Corpus Callosum | 0.00063 (9.2e-05) | 0.00057 (9.8e-05) | 1 | 8.514e-08 | 5000 | **0.0010***** | **0.0450*** |
| *Note:* (*) p < .05, (**) p < .01, (***) p < .001. Multiple comparison correction test (FWER) was applied for p-adjust values. MSS: Mean sum of squares | | | | | | | |

**Table S6.** Permutational ANCOVA cortical thickness results between African-Americans and Whites adjusting for *age, sex, education* and *economic income* from the paired subsample.

| Brain  Region  (mm) | **African-Americans**   N = 56  Mean (SD) | **Whites**   N = 56  Mean (SD) | df | MSS | Iteration | p-value | p-adjust value |
| --- | --- | --- | --- | --- | --- | --- | --- |
| Banks of superior temporal sulcus, L | 2.652 (0.1345) | 2.666 (0.1759) | 1 | 0.01138 | 115 | 0.4696 | 1.0000 |
| Banks of superior temporal sulcus, R | 2.744 (0.1603) | 2.825 (0.1147) | 1 | 0.19245 | 5000 | **2e-16***** | **0.0000***** |
| Caudal Anterior Cingulate Cortex, L | 2.742 (0.1818) | 2.663 (0.2113) | 1 | 0.12504 | 1339 | 0.0702 | 1.0000 |
| Caudal Anterior Cingulate Cortex, R | 2.536 (0.2326) | 2.506 (0.2427) | 1 | 0.03914 | 255 | 0.2824 | 1.0000 |
| Caudal Middle Frontal Gyrus, L | 2.762 (0.1260) | 2.719 (0.1462) | 1 | 0.02068 | 83 | 0.5542 | 1.0000 |
| Caudal Middle Frontal Gyrus, R | 2.777 (0.12289) | 2.736 (0.09964) | 1 | 0.02751 | 884 | 0.1018 | 1.0000 |
| Cuneus Cortex, L | 2.040 (0.1011) | 2.109 (0.1478) | 1 | 0.15129 | 5000 | **2e-16***** | **0.0000***** |
| Cuneus Cortex, R | 2.042 (0.1147) | 2.106 (0.1036) | 1 | 0.11114 | 5000 | **0.0084**** | 0.4788 |
| Entorhinal Cortex, L | 3.271 (0.2034) | 3.297 (0.2573) | 1 | 0.01223 | 51 | 0.7843 | 1.0000 |
| Entorhinal Cortex, R | 3.313 (0.2290) | 3.463 (0.2274) | 1 | 0.47066 | 5000 | **0.0024**** | 0.1488 |
| Frontal Pole, L | 2.870 (0.2009) | 2.788 (0.2336) | 1 | 0.11609 | 598 | 0.1438 | 1.0000 |
| Frontal Pole, R | 2.901 (0.2236) | 2.824 (0.2109) | 1 | 0.06515 | 1310 | 0.0709 | 1.0000 |
| Fusiform Gyrus, L | 2.850 (0.09736) | 2.859 (0.15727) | 1 | 0.00599 | 117 | 0.4615 | 1.0000 |
| Fusiform Gyrus, R | 2.860 (0.1161) | 2.896 (0.1133) | 1 | 0.03461 | 1045 | 0.0880 | 1.0000 |
| Inferior Parietal Cortex, L | 2.552 (0.1017) | 2.583 (0.1366) | 1 | 0.03069 | 51 | 0.7254 | 1.0000 |
| Inferior Parietal Cortex, R | 2.618 (0.09668) | 2.663 (0.09938) | 1 | 0.06623 | 5000 | **0.0088**** | 0.4928 |
| Inferior Temporal Gyrus, L | 2.930 (0.1036) | 2.912 (0.1602) | 1 | 0.00093 | 51 | 0.8627 | 1.0000 |
| Inferior Temporal Gyrus, R | 2.954 (0.1147) | 2.983 (0.1087) | 1 | 0.02607 | 858 | 0.1049 | 1.0000 |
| Insula, L | 3.020 (0.1444) | 3.053 (0.1763) | 1 | 0.01958 | 306 | 0.2483 | 1.0000 |
| Insula, R | 2.969 (0.1447) | 3.031 (0.1212) | 1 | 0.11991 | 5000 | **0.0052**** | 0.3068 |
| Isthmus of Cingulate Gyrus, L | 2.273 (0.2116) | 2.310 (0.3214) | 1 | 0.07354 | 51 | 0.6667 | 1.0000 |
| Isthmus of Cingulate Gyrus, R | 2.304 (0.1802) | 2.311 (0.1718) | 1 | 0.00572 | 69 | 0.5942 | 1.0000 |
| Lateral Occipital Cortex, L | 2.234 (0.1040) | 2.290 (0.1412) | 1 | 0.08767 | 5000 | **0.0120*** | 0.6600 |
| Lateral Occipital Cortex, R | 2.273 (0.1102) | 2.348 (0.1040) | 1 | 0.14341 | 5000 | **2e-16***** | **0.0000***** |
| Lateral Orbitofrontal Cortex, L | 2.789 (0.1476) | 2.807 (0.1446) | 1 | 0.02342 | 828 | 0.1087 | 1.0000 |
| Lateral Orbitofrontal Cortex, R | 2.808 (0.1417) | 2.821 (0.1242) | 1 | 0.00929 | 227 | 0.3084 | 1.0000 |
| Lingual Gyrus, L | 2.125 (0.09406) | 2.162 (0.12492) | 1 | 0.03992 | 51 | 0.8824 | 1.0000 |
| Lingual Gyrus, R | 2.132 (0.10750) | 2.197 (0.09829) | 1 | 0.08365 | 5000 | **0.0068**** | 0.3944 |
| Medial Orbitofrontal Cortex, L | 2.619 (0.1388) | 2.543 (0.1550) | 1 | 0.10103 | 5000 | 0.0591 | 0.4309 |
| Medial Orbitofrontal Cortex, R | 2.774 (0.1516) | 2.742 (0.1342) | 1 | 0.01387 | 84 | 0.5476 | 1.0000 |
| Middle Temporal Gyrus, L | 2.950 (0.1184) | 2.974 (0.1461) | 1 | 0.02986 | 764 | 0.1165 | 1.0000 |
| Middle Temporal Gyrus, R | 3.013 (0.1111) | 3.070 (0.1032) | 1 | 0.10818 | 5000 | **0.0004***** | **0.0256*** |
| Paracentral Sulcus, L | 2.542 (0.1193) | 2.565 (0.1577) | 1 | 0.01968 | 365 | 0.2164 | 1.0000 |
| Paracentral Sulcus, R | 2.588 (0.1187) | 2.599 (0.1431) | 1 | 0.00402 | 130 | 0.4385 | 1.0000 |
| Parahippocampal Gyrus, L | 2.707 (0.2213) | 2.755 (0.2896) | 1 | 0.12763 | 795 | 0.1119 | 1.0000 |
| Parahippocampal Gyrus, R | 2.656 (0.1573) | 2.725 (0.2558) | 1 | 0.13912 | 803 | 0.1108 | 1.0000 |
| Parsopercularis, L | 2.796 (0.1117) | 2.746 (0.1571) | 1 | 0.04624 | 898 | 0.1002 | 1.0000 |
| Parsopercularis, R | 2.854 (0.1314) | 2.831 (0.1010) | 1 | 0.00470 | 84 | 0.5476 | 1.0000 |
| Parsorbitalis, L | 2.790 (0.1663) | 2.762 (0.1583) | 1 | 0.00369 | 211 | 0.3222 | 1.0000 |
| Parsorbitalis, R | 2.843 (0.1390) | 2.836 (0.1417) | 1 | 0.00005 | 51 | 0.9607 | 1.0000 |
| Parstriangularis, L | 2.634 (0.1437) | 2.594 (0.1374) | 1 | 0.02157 | 114 | 0.4737 | 1.0000 |
| Parstriangularis, R | 2.703 (0.13813) | 2.685 (0.09222) | 1 | 0.00004 | 51 | 0.9803 | 1.0000 |
| Pericalcarine Cortex, L | 1.978 (0.1141) | 2.011 (0.1348) | 1 | 0.04908 | 1549 | 0.0606 | 1.0000 |
| Pericalcarine Cortex, R | 1.982 (0.12583) | 2.022 (0.09597) | 1 | 0.04574 | 990 | 0.0919 | 1.0000 |
| Postcentral Gyrus, L | 2.176 (0.09014) | 2.226 (0.12314) | 1 | 0.07804 | 5000 | **0.0048**** | 0.2880 |
| Postcentral Gyrus, R | 2.204 (0.1009) | 2.260 (0.1091) | 1 | 0.08961 | 5000 | **0.0036**** | 0.2196 |
| Posterior Cingulate Gyrus, L | 2.582 (0.1247) | 2.582 (0.1440) | 1 | 0.00247 | 202 | 0.3317 | 1.0000 |
| Posterior Cingulate Gyrus, R | 2.511 (0.1506) | 2.553 (0.1572) | 1 | 0.06181 | 102 | 0.5000 | 1.0000 |
| Precentral Gyrus, L | 2.74 (0.09945) | 2.72 (0.14836) | 1 | 0.00434 | 115 | 0.4696 | 1.0000 |
| Precentral Gyrus, R | 2.739 (0.1121) | 2.725 (0.1123) | 1 | 0.00383 | 51 | 0.6863 | 1.0000 |
| Precuneus Cortex, L | 2.514 (0.1153) | 2.532 (0.1408) | 1 | 0.01472 | 440 | 0.1864 | 1.0000 |
| Precuneus Cortex, R | 2.543 (0.1150) | 2.578 (0.1091) | 1 | 0.03974 | 327 | 0.2355 | 1.0000 |
| Rostral Anterior Cingulate Cortex, L | 3.081 (0.1964) | 3.015 (0.2433) | 1 | 0.07766 | 328 | 0.2348 | 1.0000 |
| Rostral Anterior Cingulate Cortex, R | 3.056 (0.1737) | 2.976 (0.1898) | 1 | 0.11732 | 512 | 0.1640 | 1.0000 |
| Rostral Middle Frontal Gyrus, L | 2.584 (0.1360) | 2.550 (0.1138) | 1 | 0.01359 | 197 | 0.3401 | 1.0000 |
| Rostral Middle Frontal Gyrus, R | 2.600 (0.13226) | 2.576 (0.09665) | 1 | 0.01565 | 349 | 0.7647 | 1.0000 |
| Superior Frontal Gyrus, L | 2.869 (0.1321) | 2.834 (0.1498) | 1 | 0.02183 | 574 | 0.1498 | 1.0000 |
| Superior Frontal Gyrus, R | 2.897 (0.1461) | 2.869 (0.1186) | 1 | 0.00994 | 84 | 0.5476 | 1.0000 |
| Superior Parietal Cortex, L | 2.281 (0.1039) | 2.294 (0.1276) | 1 | 0.00946 | 51 | 0.9019 | 1.0000 |
| Superior Parietal Cortex, R | 2.316 (0.1015) | 2.339 (0.1031) | 1 | 0.02009 | 918 | 0.0991 | 1.0000 |
| Superior Temporal Gyrus, L | 2.888 (0.1137) | 2.860 (0.1600) | 1 | 0.01329 | 90 | 0.5333 | 1.0000 |
| Superior Temporal Gyrus, R | 2.942 (0.1237) | 2.934 (0.1203) | 1 | 0.00011 | 51 | 1.0000 | 1.0000 |
| Supramarginal Gyrus, L | 2.633 (0.1116) | 2.660 (0.1373) | 1 | 0.02682 | 653 | 0.1332 | 1.0000 |
| Supramarginal Gyrus, R | 2.668 (0.1017) | 2.726 (0.1158) | 1 | 0.11242 | 5000 | **2e-16***** | **0.0000***** |
| Temporal Pole, L | 2.870 (0.2009) | 2.788 (0.2336) | 1 | 0.00335 | 51 | 0.8431 | 1.0000 |
| Temporal Pole, R | 3.588 (0.2633) | 3.648 (0.2882) | 1 | 0.06347 | 304 | 0.2500 | 1.0000 |
| Transverse Temporal Cortex, L | 2.598 (0.1683) | 2.656 (0.2167) | 1 | 0.15151 | 563 | 0.1510 | 1.0000 |
| Transverse Temporal Cortex, R | 2.653 (0.1645) | 2.763 (0.1619) | 1 | 0.34883 | 5000 | **0.0016**** | 0.1008 |
| *Note:* (*) p < .05, (**) p < .01, (***) p < .001. Multiple comparison correction test (FWER) was applied for p-adjust values. MSS: Mean sum of squares | | | | | | | |

**Table S7.** Permutational ANCOVA surface cortical area results between African-Americans and Whites adjusting for *age, sex, education* and *economic income* from the paired subsample.

| Brain  Region  (mm^2^) | **African-Americans**   N = 56  Mean (SD) | **Whites**   N = 56  Mean (SD) | df | MSS | Iteration | p-value | p-adjust value |
| --- | --- | --- | --- | --- | --- | --- | --- |
| Banks of superior temporal sulcus, L | 1053.9 (181.69) | 1044.4 (193.98) | 1 | 8151 | 183 | 0.3552 | 1.0000 |
| Banks of superior temporal sulcus, R | 951.62 (144.70) | 959.14 (175.71) | 1 | 678 | 51 | 0.7843 | 1.0000 |
| Caudal Anterior Cingulate Cortex, L | 691.21 (171.19) | 645.62 (149.40) | 1 | 87764 | 1079 | 0.0852 | 1.0000 |
| Caudal Anterior Cingulate Cortex, R | 790.23 (214.64) | 800.25 (190.76) | 1 | 371 | 51 | 0.8627 | 1.0000 |
| Caudal Middle Frontal Gyrus, L | 2227.3 (411.95) | 2316.5 (385.36) | 1 | 168488 | 504 | 0.1667 | 1.0000 |
| Caudal Middle Frontal Gyrus, R | 2031.7 (430.70) | 2166.0 (374.76) | 1 | 349660 | 603 | 0.1426 | 1.0000 |
| Cuneus Cortex, L | 1379.7 (213.90) | 1471.6 (277.79) | 1 | 109952 | 156 | 0.3910 | 1.0000 |
| Cuneus Cortex, R | 1404.1 (203.64) | 1524.9 (297.19) | 1 | 349406 | 5000 | **0.0186*** | 1.0000 |
| Entorhinal Cortex, L | 392.04 (79.933) | 418.48 (87.646) | 1 | 21162 | 858 | 0.1049 | 1.0000 |
| Entorhinal Cortex, R | 340.77 (67.162) | 338.80 (70.712) | 1 | 168 | 5 | 0.7843 | 1.0000 |
| Frontal Pole, L | 195.09 (33.976) | 205.27 (37.527) | 1 | 1305.2 | 51 | 0.8431 | 1.0000 |
| Frontal Pole, R | 255.23 (41.278) | 278.38 (44.632) | 1 | 11247.3 | 5000 | **0.0126*** | 0.7812 |
| Fusiform Gyrus, L | 3241.1 (494.38) | 3284.0 (468.98) | 1 | 9 | 51 | 1.0000 | 1.0000 |
| Fusiform Gyrus, R | 3091.1 (409.40) | 3222.1 (487.21) | 1 | 225898 | 149 | 0.4027 | 1.0000 |
| Inferior Parietal Cortex, L | 4555.7 (697.13) | 4581.1 (719.89) | 1 | 14639 | 54 | 0.8235 | 1.0000 |
| Inferior Parietal Cortex, R | 5466.6 (756.46) | 5407.3 (796.12) | 1 | 286843 | 87 | 0.5402 | 1.0000 |
| Inferior Temporal Gyrus, L | 3151.9 (459.97) | 3407.9 (485.12) | 1 | 1190633 | 5000 | **0.0050**** | 0.3200 |
| Inferior Temporal Gyrus, R | 3074.5 (461.85) | 3203.9 (442.37) | 1 | 354570 | 51 | 1.0000 | 1.0000 |
| Insula, L | 2185.0 (256.33) | 2221.1 (269.44) | 1 | 8601 | 51 | 0.7059 | 1.0000 |
| Insula, R | 2321.9 (310.78) | 2348.1 (307.35) | 1 | 9387 | 51 | 1.0000 | 1.0000 |
| Isthmus of Cingulate Gyrus, L | 1039.6 (396.80) | 1046.1 (318.34) | 1 | 1281 | 51 | 1.0000 | 1.0000 |
| Isthmus of Cingulate Gyrus, R | 947.80 (158.11) | 949.91 (160.81) | 1 | 28 | 51 | 1.0000 | 1.0000 |
| Lateral Occipital Cortex, L | 4432.8 (606.08) | 4688.7 (619.19) | 1 | 1855172 | 5000 | **0.0026**** | 0.1716 |
| Lateral Occipital Cortex, R | 4281.6 (629.35) | 4596.2 (582.93) | 1 | 2123586 | 5000 | **0.0012**** | 0.0816 |
| Lateral Orbitofrontal Cortex, L | 2511.6 (288.72) | 2601.8 (342.02) | 1 | 66222 | 306 | 0.24837 | 1.0000 |
| Lateral Orbitofrontal Cortex, R | 2486.0 (289.36) | 2549.9 (312.51) | 1 | 35982 | 51 | 0.8824 | 1.0000 |
| Lingual Gyrus, L | 2863.0 (396.94) | 3081.1 (395.55) | 1 | 988306 | 5000 | **0.0022**** | 0.1474 |
| Lingual Gyrus, R | 2956.6 (423.85) | 3089.2 (424.60) | 1 | 374795 | 1299 | 0.0715 | 1.0000 |
| Medial Orbitofrontal Cortex, L | 1927.6 (329.58) | 1970.0 (295.60) | 1 | 11472 | 174 | 0.3678 | 1.0000 |
| Medial Orbitofrontal Cortex, R | 1773.0 (219.82) | 1788.9 (245.13) | 1 | 64 | 51 | 1.0000 | 1.0000 |
| Middle Temporal Gyrus, L | 2960.1 (441.08) | 3081.3 (439.63) | 1 | 252304 | 550 | 0.1545 | 1.0000 |
| Middle Temporal Gyrus, R | 3306.1 (413.05) | 3450.5 (483.69) | 1 | 364785 | 600 | 0.1433 | 1.0000 |
| Paracentral Sulcus, L | 1313.4 (164.92) | 1309.3 (189.19) | 1 | 1227 | 51 | 0.9020 | 1.0000 |
| Paracentral Sulcus, R | 1520.8 (249.46) | 1507.3 (238.96) | 1 | 10792 | 81 | 0.5556 | 1.0000 |
| Parahippocampal Gyrus, L | 718.96 (117.08) | 706.38 (125.51) | 1 | 8041 | 256 | 0.2812 | 1.0000 |
| Parahippocampal Gyrus, R | 685.07 ( 90.185) | 684.02 (114.723) | 1 | 142 | 55 | 0.6545 | 1.0000 |
| Parsopercularis, L | 1589.2 (246.49) | 1742.3 (303.20) | 1 | 427219 | 4301 | **0.0227*** | 1.0000 |
| Parsopercularis, R | 1292.1 (226.98) | 1418.9 (233.89) | 1 | 349039 | 5000 | **0.0028**** | 0.1820 |
| Parsorbitalis, L | 613.18 (78.176) | 629.55 (81.233) | 1 | 651.5 | 121 | 0.4545 | 1.0000 |
| Parsorbitalis, R | 745.05 ( 95.406) | 783.48 (112.394) | 1 | 17390.6 | 725 | 0.1214 | 1.0000 |
| Parstriangularis, L | 1273.7 (212.37) | 1311.0 (230.09) | 1 | 5481 | 51 | 0.8627 | 1.0000 |
| Parstriangularis, R | 1520.7 (239.65) | 1527.8 (257.68) | 1 | 13730 | 51 | 0.7451 | 1.0000 |
| Pericalcarine Cortex, L | 1385.7 (264.15) | 1517.7 (279.10) | 1 | 379473 | 5000 | **0.0124*** | 0.7 |
| Pericalcarine Cortex, R | 1550.4 (263.25) | 1661.1 (278.88) | 1 | 274308 | 2925 | **0.0331*** | 1.0000 |
| Postcentral Gyrus, L | 3998.8 (517.21) | 4056.2 (572.38) | 1 | 32563 | 51 | 1.0000 | 1.0000 |
| Postcentral Gyrus, R | 3881.0 (535.20) | 3915.9 (528.94) | 1 | 25 | 51 | 1.0000 | 1.0000 |
| Posterior Cingulate Gyrus, L | 1201.6 (152.93) | 1175.4 (206.50) | 1 | 40888 | 494 | 0.1700 | 1.0000 |
| Posterior Cingulate Gyrus, R | 1187.1 (169.30) | 1244.6 (208.23) | 1 | 41749 | 121 | 0.4545 | 1.0000 |
| Precentral Gyrus, L | 4599.2 (549.39) | 4762.3 (823.82) | 1 | 512068 | 352 | 0.2216 | 1.0000 |
| Precentral Gyrus, R | 4691.1 (549.05) | 4869.9 (674.70) | 1 | 651545 | 1405 | 0.0669 | 1.0000 |
| Precuneus Cortex, L | 3610.7 (519.09) | 3764.1 (475.09) | 1 | 353156 | 315 | 0.2412 | 1.0000 |
| Precuneus Cortex, R | 3770.2 (527.04) | 3970.3 (633.84) | 1 | 596318 | 2258 | **0.0425*** | 1.0000 |
| Rostral Anterior Cingulate Cortex, L | 5810.5 (796.10) | 5976.8 (861.81) | 1 | 3669 | 55 | 0.6545 | 1.0000 |
| Rostral Anterior Cingulate Cortex, R | 630.77 (148.74) | 670.09 (153.44) | 1 | 7382 | 69 | 0.5942 | 1.0000 |
| Rostral Middle Frontal Gyrus, L | 5810.5 (796.10) | 5976.8 (861.81) | 1 | 188303 | 51 | 0.8431 | 1.0000 |
| Rostral Middle Frontal Gyrus, R | 6039.8 ( 836.83) | 6152.8 (1012.47) | 1 | 102049 | 51 | 0.7451 | 1.0000 |
| Superior Frontal Gyrus, L | 6931.2 (868.67) | 7284.8 (913.00) | 1 | 2271114 | 869 | 0.1036 | 1.0000 |
| Superior Frontal Gyrus, R | 6849.0 (828.76) | 7138.8 (898.47) | 1 | 1453927 | 894 | 0.1007 | 1.0000 |
| Superior Parietal Cortex, L | 5080.8 (565.34) | 5371.2 (788.03) | 1 | 1371913 | 2220 | **0.0432*** | 1.0000 |
| Superior Parietal Cortex, R | 5100.6 (558.74) | 5383.9 (779.69) | 1 | 1070639 | 1960 | **0.0489*** | 1.0000 |
| Superior Temporal Gyrus, L | 3760.1 (455.16) | 3738.7 (493.40) | 1 | 112105 | 159 | 0.3899 | 1.0000 |
| Superior Temporal Gyrus, R | 3600.5 (413.43) | 3581.9 (409.29) | 1 | 95084 | 353 | 0.2209 | 1.0000 |
| Supramarginal Gyrus, L | 3767.0 (554.73) | 3727.9 (592.74) | 1 | 165230 | 206 | 0.3301 | 1.0000 |
| Supramarginal Gyrus, R | 3617.4 (589.36) | 3607.3 (633.01) | 1 | 63123 | 88 | 0.5341 | 1.0000 |
| Temporal Pole, L | 477.41 (60.350) | 495.98 (69.199) | 1 | 7326 | 309 | 0.2460 | 1.0000 |
| Temporal Pole, R | 436.43 (69.392) | 421.05 (62.057) | 1 | 9498.5 | 251 | 0.2869 | 1.0000 |
| Transverse Temporal Cortex, L | 445.77 (76.559) | 445.91 (98.018) | 1 | 2872 | 51 | 1.0000 | 1.0000 |
| Transverse Temporal Cortex, R | 332.07 (58.545) | 323.57 (58.445) | 1 | 6914 | 1588 | 0.0598 | 1.0000 |
| *Note:*  (*) p < .05, (**) p < .01. Multiple comparison correction test (FWER) was applied for p-adjust values. MSS: Mean sum of squares | | | | | | | |
